# Revision of the *Schistura cincticauda* species group (Teleostei, Nemacheilidae) using molecular and morphological markers

**DOI:** 10.1038/s41598-023-42852-1

**Published:** 2023-10-09

**Authors:** Tomáš Dvořák, Jörg Bohlen, Maurice Kottelat, Vendula Šlechtová

**Affiliations:** 1 Institute of Animal Physiology and Genetics, Czech Academy of Sciences, Rumburská 89, 277 21 Liběchov, Czech Republic; 2 Department of Zoology, Faculty of Science, Charles University, Viničná 7, 128 00 Prague 2, Czech Republic; 3 Delémont, Switzerland; 4 Lee Kong Chian Natural History Museum, National University of Singapore, 2 Conservatory Drive, Singapore, 117377 Singapore

**Keywords:** Evolution, Genetics, Zoology

## Abstract

To approach the taxonomy of large and complex animal groups it is of advantage to focus on species groups with shared derived character state. We investigate the composition, morphological characteristics and relationships of and within the *Schistura cincticauda* species group, whose members are small freshwater fishes that inhabit streams and rivers in eastern Myanmar and western and southern Thailand. A phylogenetic analysis using molecular genetic markers demonstrated the monophyly of this group; a combined genetic and morphological analysis revealed the inclusion of at least twelve species. They share the presence of a pair of black marks on the lower lip, one on each side of the median interruption (these marks may be reduced to few melanophores or even missing in some individuals). Additionally, all species share a small body size (max. 60 mm SL), an incomplete lateral line reaching at most to vertical through anal-fin base, and the absence of sexual dimorphism. Each of the 12 species is diagnosed by a unique combination of character states in fin ray numbers, anus position, presence/absence of an axillary pelvic lobe, and colour pattern. The distribution areas of several species overlap and five cases of syntopic occurrence are known. Five unnamed species are described herein.

## Introduction

The freshwater fish family Nemacheilidae inhabits nearly the whole of Eurasia, and with > 700 species in about 50 genera is one of the largest in Eurasia^1,2^. However, still new species and genera are regularly described^1,3–9^.

**Figure 1:**
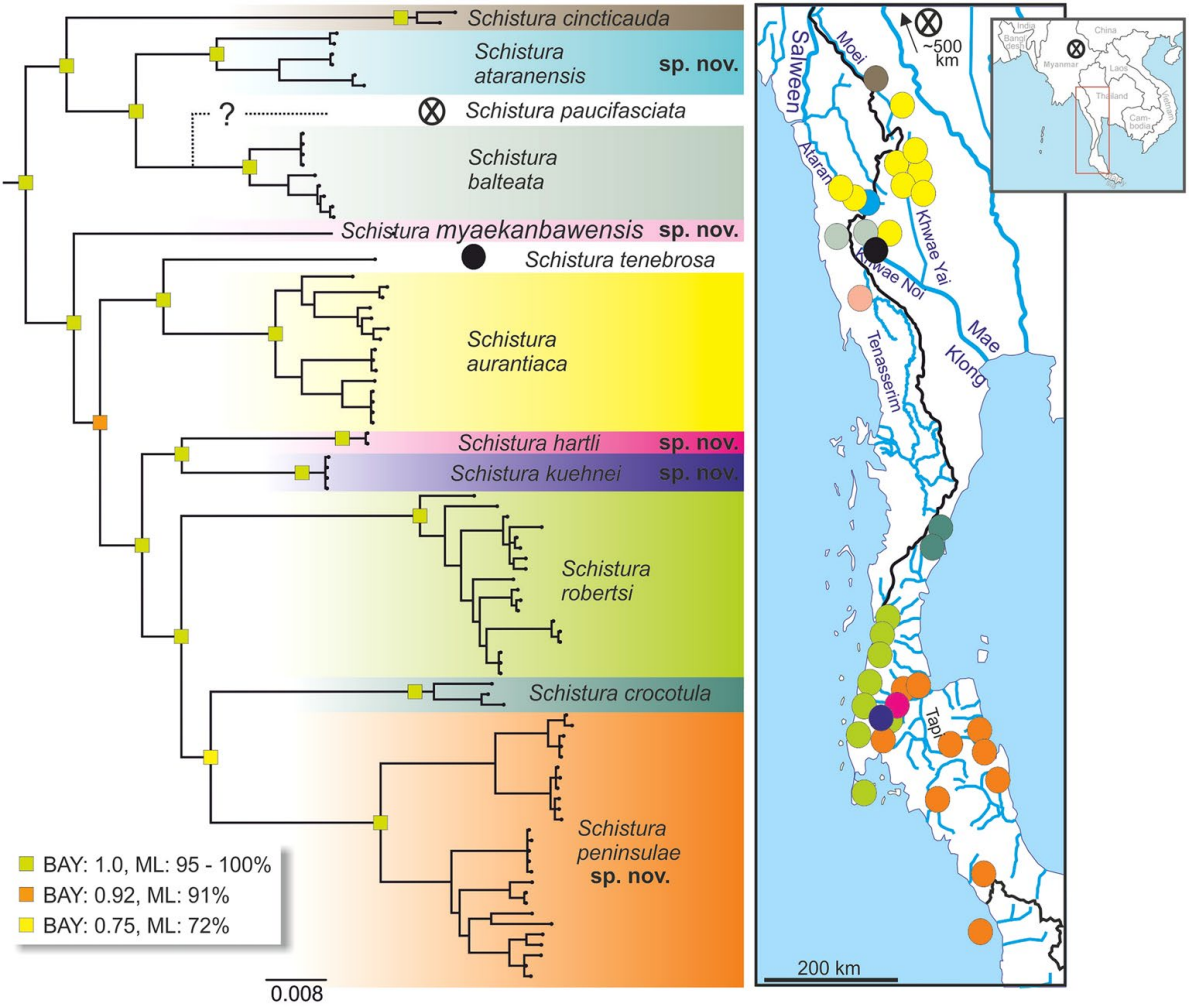
Bayesian tree of the concatenated dataset (mitochondrial cytochrome b plus nuclear IRBP 2 genes) showing the phylogenetic relationships of the species of the *Schistura cincticauda* species group and the proposed position of *S. paucifasciata* as concluded from its shared rare pigmentation element with *S. balteata*. The Bayesian (BAY) and maximum likelihood (ML) analyses revealed congruent topologies; the rectangles at the nodes indicate the posterior probabilities and bootstrap support. The map indicates the geographic origin of the analysed samples of the species. Colours in the tree correspond to colours in map; the values at the nodes represent the posterior possibilities.

With > 230 described species, the genus *Schistura* is the largest genus of Nemacheilidae, and it is distributed across most of South and Southeast Asia^2,10^. It is well known that *Schistura* is a polyphyletic assemblage, a ‘catch-all genus’^2,4,11–14^, but the high number of species and the wide distribution have hampered a global revision of the genus. Few researchers have tried to identify natural groups of related species of manageable size within the genus in order to analyse their taxonomy and, biogeography, and to contribute to their conservation. This is an unfortunate situation and experience shows that it is feasible to distinguish a number of groups of species that share distinctive morphological feature^11,15,16^. It is predictable that several such species groups might once be recognised as distinct genera. In the present study we focus on the *Schistura cincticauda* species group, which is distributed in eastern Myanmar and western and southern Thailand (Fig. 1). The existence of a species group made of *S. cincticauda* and related species was first noted, based on morphological data, by Kottelat^11^ and more recently defined by genetic data^15^. This recent analysis found ten species in the group, each well supported by mitochondrial and nuclear data. In this genetic study, the group was referred to as the ‘*Schistura robertsi* group’, it is more appropriate, following practices in ichthyology, to use the name of the oldest member, *S. cincticauda*, as name for the group.

Most species of the *S. cincticauda* species group inhabit very small to medium streams. Some species like *S. ataranensis*, *S. aurantiaca* and *S. balteata* are found mainly in the upper reaches of streams. The smallest species (*S. hartli, S. kuehnei, S. peninsulae*, *S. robertsi*) are regularly found in very small forest streams, in shallow water, among leave litter. These headwater habitats have a high degree of isolation from each other, and are occupied by very small fish populations. Therefore the impact of genetic drift and local adaptation can be expected to be high in the *S. cincticauda* species group, leading to a fast and geographically small-scaled evolution. The present study aims to test the monophyly of the *S. cincticauda* group with a larger number of species, to provide a morphological diagnosis, identify the species belonging to the group, review their morphology and describe the unnamed species.

## Methods

About 300 specimens of the *S. cincticauda* group were examined, including the complete set of specimens analysed by Bohlen et al.^15^. Specimens were either fixed in 4% formaldehyde and stored in 70% ethanol or fixed and stored in 96% ethanol. Method for measurements and counts follow Kottelat^11^. The phylogenetic trees have been recalculated using the dataset from Bohlen et al.^15^ plus the cytochrome b sequence of *S. tenebrosa* (GenBank accession number JQ659026) and sequences of 54 species of *Schistura* and of 23 species from other nemacheilid genera from GenBank (Supplementary Material Table S1). Method of Bayesian analysis, StarBEAST species tree calculation and maximum likelihood tree reconstruction followed Bohlen et al.^15^. Genetically analysed specimens of the ingroup were grouped into their clades and searched for inter-clade differences in morphology. Candidate characters were evaluated and checked on the non-sequenced specimens. For details of morp﻿hological characters for the new species see Supplementary material. Collection abbreviations: BMNH, Natural History Museum, London, UK; CMK, Collection of Maurice Kottelat, Delémont, Switzerland; IAPG, Collection of the Institute of Animal Physiology and Genetics, Liběchov, Czech Republic; MCZ, Museum of Comparative Zoology, Harward University, Cambridge, USA; MHNG, Muséum d'Histoire Naturelle, Geneva, Switzerland; UF, University of Florida, Gainsville, USA; ZRC Zoological Reference Collection, Lee Kong Chian Natural History Museum, National University of Singapore, Singapore. The publication is registered in ZooBank under the number LSID urn:lsid:zoobank.org:pub:431013B9-DF21-4078-BFE8-C7BE622492D5.

## Results and discussion

### The *Schistura cincticauda* species group

**Figure 2:**
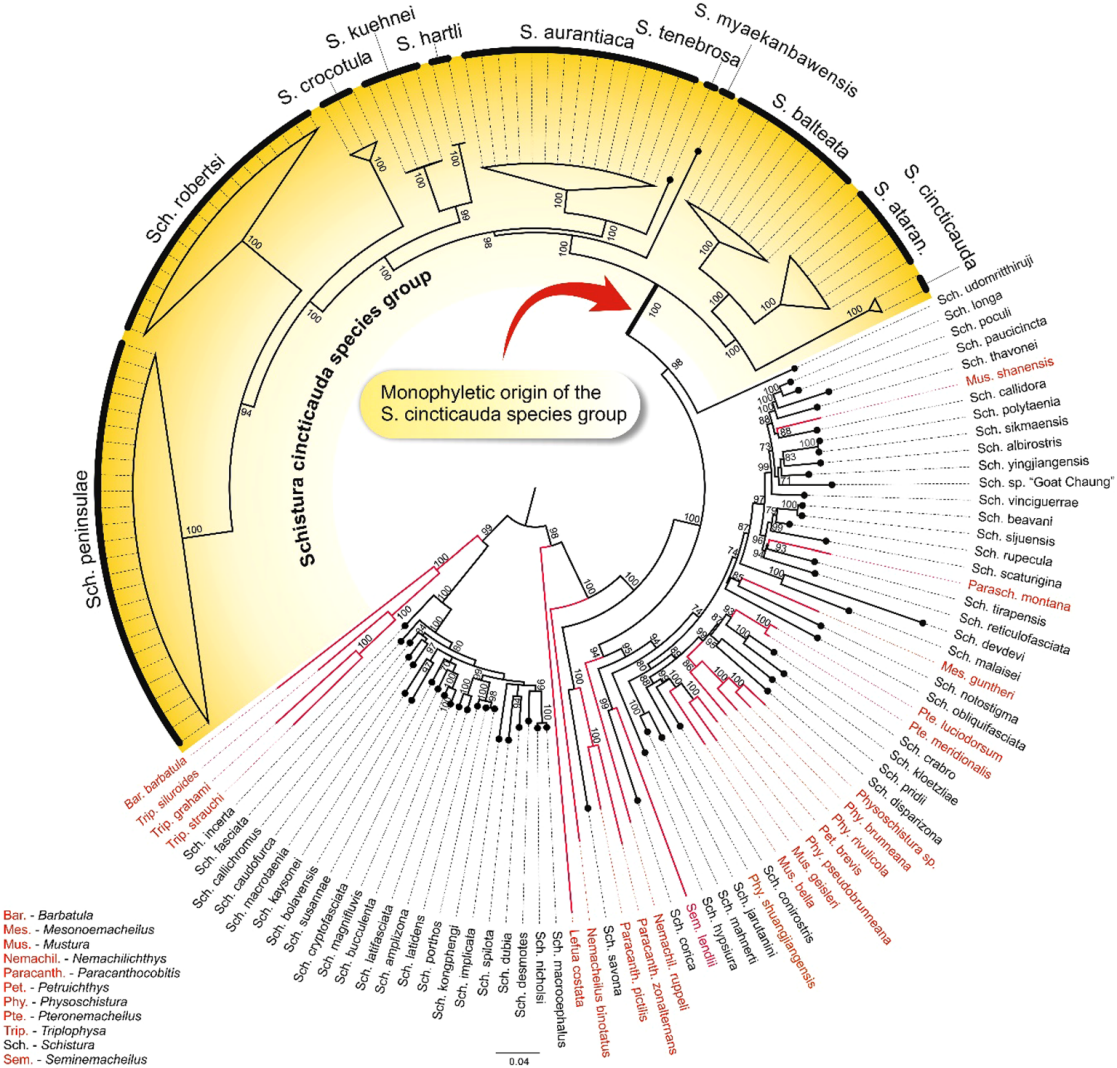
Maximum Likelihood phylogenetic tree showing the monophyly of the *Schistura cincticauda* species group (indicated by red arrow). Besides the 11 species of the *Schistura cincticauda* group for which genetic data are available (underlaid with yellow colour), the analysis covered 54 additional species of the genus *Schistura* (species names and branches in black) and 23 species of other nemacheilid genera (species names and branches in red). The tree was rooted with *Cobitis taenia*. The values at the nodes represent relevant statistical supports in percentages of 5000 bootstrap replicates. Values lower than 70% are not shown.

The reconstructed phylogeny including 11 species of the *S. cincticauda* species group plus 77 other species of Nemacheilidae demonstrated the monophyly of the *S. cincticauda* species group (Fig. 2). The combined results from genetic and morphological analyses show that the *S. cincticauda* group contains at least 12 species, seven of them with a valid name [*S. aurantiaca* Plongsesthee et al., 2011, *S. balteata* (Rendahl, 1948), *S. cincticauda* (Blyth, 1860), *S. crocotula* Plongsesthee et al., 2013, *S. paucifasciata* (Hora 1929), *S. robertsi* Kottelat, 1990 and *S. tenebrosa* Kangran et al., 2012] and five unnamed species. Genetic data were available for all but one (*S. paucifasciata*) of these species.

**Figure 3:**
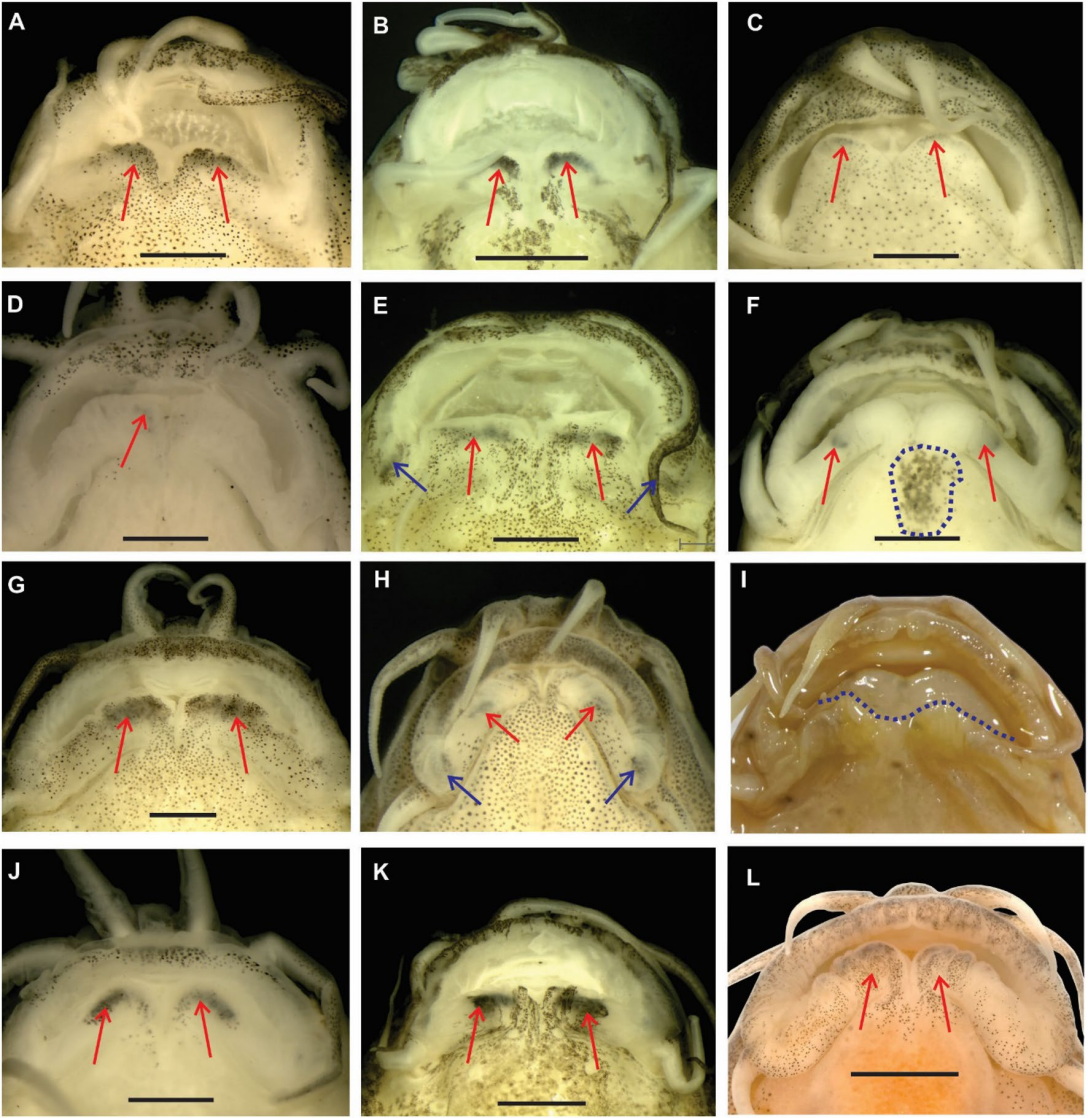
Ventral view of the mouth of the twelve species presently assigned to the *Schistura cincticauda* species group. Red arrows indicate the characteristic pair of black blotches on the lower lip, blue arrows indicate an additional pair of black blotches on the lower lip. (**A**) *S. ataranensis*, ZRC 61579, holotype, 43.8 mm SL; (**B**) *S. aurantiaca*, IAPG A11272-11,274, 27.0 mm SL; (**C**) *S. balteata*, IAPG A2554, 32.2 mm SL; (**D**) *S. cincticauda,* IAPG A8313, 30.0 mm SL; (**E**) *S. crocotula*, IAPG A10513-10,517, 35.5 mm SL; (**F**) *S. hartli,* CMK 28810, paratype, 36.6 mm SL, blue outline depicts the diagnostic black mark posterior of the median incision; (**G**) *S. kuehnei*, ZRC 61582, holotype, 37.1 mm SL; (**H**) *S. myaekanbawensis*, CMK 24993, paratype, 29.4 mm SL; (**I**) *S. paucifasciata*, BMNH 930.3.3.4, paratype, 46.6 mm SL, blue dotted line shows anterior margin of lower lip; (**J**) *S. peninsulae*, IAPG A11506, 33.8 mm SL; (**K**) *S. robertsi*, IAPG A10967, 31.4 mm SL; (**L**) *S. tenebrosa*, UF 181418, paratype, 33.2 mm SL.

Externally, the species of the group share one synapomorphy: the presence of two black marks on the lower lip, one on each side of the median interruption (Fig. 3). These marks are located in a deeper layer of the skin than the general pigmentation of the head and are always black, while the general head and body pigmentation can be dark grey or brown. While the general head and body pigmentation may fade when the live fish is stressed, or change during fixation or after long time storage, the black marks on the lower lip prove to be much more stable and are visible in nearly all of about 300 analysed specimens, including specimens preserved more than 50 years ago. The stability of the black marks on the lower lip resembles that of the black marks on the base of the caudal fin or on the first rays of the dorsal fin in most species of Nemacheilidae, which is known to survive in many museum specimens, including material collected in the early nineteenth century (MK, pers. observ.).

The position of the black marks on the lower lip varies between species of the group, ranging from close to the median interruption of the lip (*S. peninsulae*; Fig. 3J) to almost halfway between median interruption and corner of mouth (*S. hartli*; Fig. 3F). The marks vary in size and intensity: in *S. cincticauda* the marks are only weakly developed (Fig. 3D); often consisting of only a few melanophores on one side of the lip and easily overlooked, while in *S. kuehnei* and *S. robertsi* they are prominent black blotches that stretch along one third of the lower-lip (Fig. 3G,K). In *S. crocotula* and *S. myaekanbawensis*, a second pair of black marks is located on the lower lip, at the corner of mouth (Fig. 3E,H). Our material of *S. balteata* originated from two localities; one in Myanmar (details not known) and one in the upper Mae Khlong basin in Thailand. While the marks are present in all four specimens from Myanmar and two specimens from the Thai locality (Fig. 3C), they are missing in the remaining four specimens from the Thai locality (not shown). The presence of black marks could not be confirmed in the only investigated specimen of *S. paucifasciata* (collected in 1911), in which the lower lip is pulled backwards (a fixation artefact or the result of desiccation, before we examined it in 1989) (Fig. 3I). We are not aware of any species of *Schistura* s.l. outside the *S. cincticauda* group with such marks on the lower lip, thus they appear to be a synapomorphy of the group. Despite their occasional absence, or difficulties to observe them, they represent the easiest external diagnostic character of the *S. cincticauda* group.

Although the black marks are not visible in the only available specimens of *S. paucifasciata* we consider this species to belong to the *cincticauda* group on the basis of the following reasons: *S. paucifasciata* shares with *S. balteata* the unusual colour pattern of having only few narrow, black, contrasting bars in the middle of the flank and the rest of the flank light brown. In both species the dorsal adipose crest on the caudal peduncle is higher and the axillary pelvic lobe larger than in other species of the group. It shares with all species of the *cincticauda* group an incomplete lateral line, a small body size and an emarginate caudal fin. Kottelat^11^ already noted that the colour pattern of juveniles (figured by Hora^17^) has some similarities with that of *S. cincticauda* (and also *S. aurantiaca* described inbetween)*.* Since *S. paucifasciata* and *S. balteata* are the only two species of *Schistura* with the above described colour pattern we consider them to represent sister species. Thus the phylogenetic position of *S. paucifasciata* is indicated in our phylogenetic reconstruction as sister to *S. balteata* (Fig. 1). The affiliation of *S. tenebrosa* to the *cincticauda* group is demonstrated by its black marks on the lower lip and genetic data.

A few other species have been suggested to be closely related to *S. cincticauda.* Kottelat^11^ listed *S. cincticauda, S. robertsi, S. daubentoni* and possibly *S. paucicincta, S. paucifasciata, S. kangjupkhulensis* and *S. malaisei*. We confirm here that *S. robertsi* and *S. paucifasciata* belong to the *S. cincticauda* species group. Our genetic data (Fig. 2) show that *S. paucicincta* and *S. malaisei* do not belong to this group. *Schistura daubentoni* does not have black marks on the lower lip; it has tubercles on the dorsal side of the pectoral fin. Both character states are not congruent with the *S. cincticauda* species group; therefore we exclude it from this group. Only figures were available of *S. kangjupkhulensis*, which did not allow to conclude on the phylogenetic position of the species; its evaluation has to await further data.

Additionally, the species of the *S. cincticauda* species group are characterised by the combination of the following features: a small size (less than 60 mm SL); an incomplete lateral line reaching at most to vertical through the anal-fin base; a relatively deep caudal peduncle with dorsal and ventral crests; a truncate or emarginate caudal fin (never forked); the anterior nostril with a flap-like tube that reaches at least to anterior margin of eye; and absence of sexual dimorphism (no morphological differences in the pectoral fins, no suborbital flap). Most species also have a comparably low number of branched rays in pectoral and caudal fin. However, although useful for identification in combination, these character states are not restricted to species of the *S. cincticauda* group, but some appear in several other species and lineages of *Schistura*.

**Figure 4:**
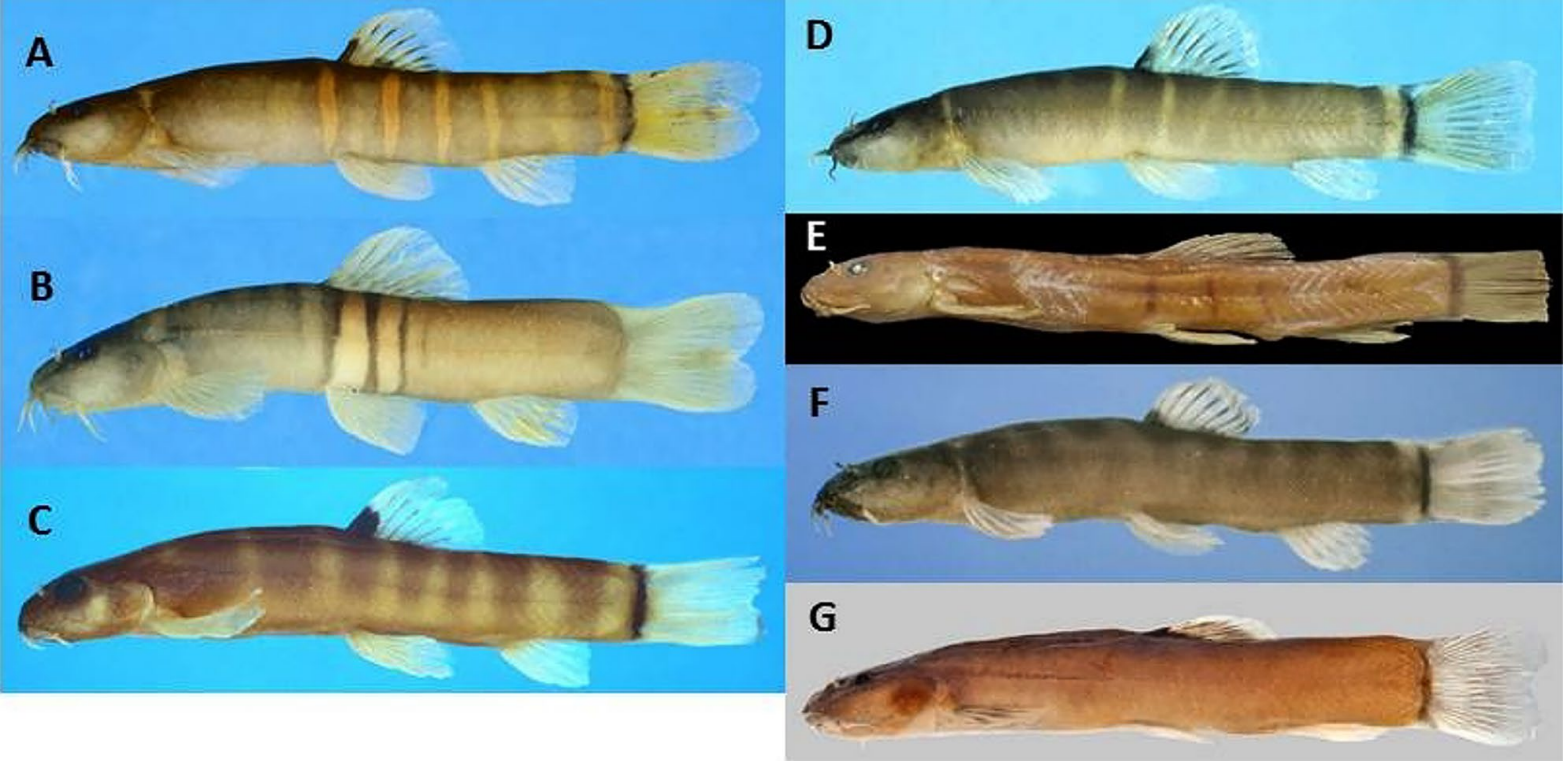
Species of the *cincticauda* species group described before 2023: (**A**) *Schistura aurantiaca*, CMK 14530, 45.8 mm SL; Thailand: Kanchanaburi province: Mae Khlong basin; (**B**) *Schistura balteata*, CMK 17236, 53.5 mm SL; Thailand: Kanchanaburi province: Mae Khlong basin: Pilok stream; (**C**) *Schistura cincticauda*, ZRC 38458, neotype, 29.3 mm SL; Thailand: Tak province: Salween basin: Moei River; (**D**) *Schistura crocotula*, CMK 16458, 30.4 mm SL; Thailand: Prachuap Khiri Khan province; (**E**) *Schistura paucifasciata*, BMNH 1930.3.3:4, paratype, 46.6 mm SL: Myanmar: Monglong; (**f**) *Schistura robertsi*, CMK 5346, paratype, 30.0 mm SL; Thailand: Phang Nga province: Phang Nga basin; (**G**) *Schistura tenebrosa*, UF 181417, holotype, 45.1 mm SL: Thailand: Kanchanaburi province: Mae Khlong drainage. (from Kangrang et al. 2012).

**Figure 5:**
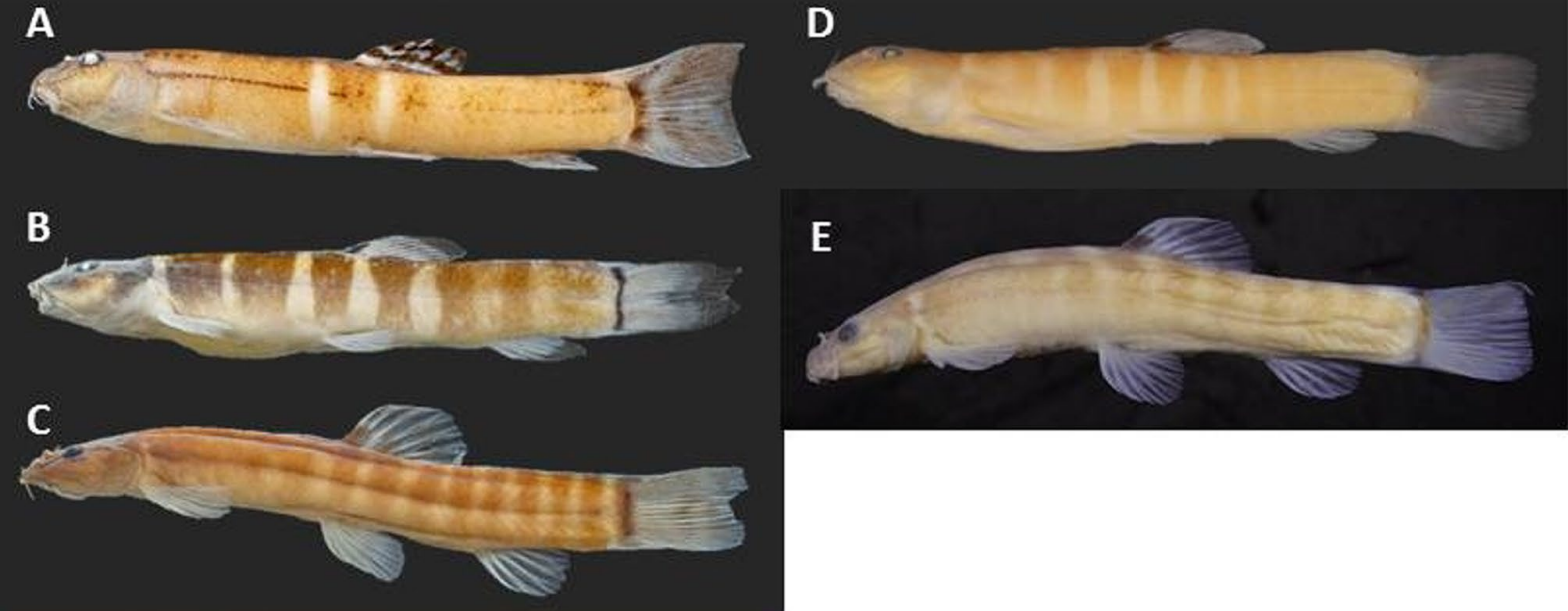
The new species described in the present study. (**A**) *Schistura ataranensis*, ZRC 61579, holotype, 43.5 mm SL; Myanmar: Kayin province: Ataran drainage; (**B**) *Schistura hartli*, ZRC 61581, holotype, 41.0 mm SL; Thailand: Surat Thani Province: Khao Sok; (**C**) *Schistura myaekanbawensis*, MHNG 2790.081, holotype, 29.8 mm SL; Myanmar: Tanintharyi Region: Tanintharyi drainage; (**D**) *Schistura kuehnei*, ZRC 61582, holotype, 37.1 mm SL; Thailand: Surat Thani Province: Tapi drainage, Khlong Sok watershed;. (**E**) *Schistura peninsulae*, ZRC 61584, holotype, 35.6 mm SL; Malaysia: Kedah province: Langkawi Island.

Most species of the *S. cincticauda* group have a prominent thick dark bar at caudal-fin base, reaching onto ventral and dorsal adipose crests. However there are exceptions; in *S. balteata* (Fig. 4B) the black bar is completely absent and in *S. kuehnei* (Fig. 5D) it is thin and interrupted in its upper third. The body colour pattern of most species of the *S. cincticauda* group consists of grey to black bars on a lighter background. In most species this pattern is already observed in juveniles. However, in *S. balteata* and *S. paucifasciata*, most bars become less sharply contrasted with growth until only 2 to 4 narrow black bars remain in the middle region of the flank, while the rest of the flank is light brown (^11^, pers. observ., Fig. 4B,E). In other species, some bars may fuse entirely, so that parts of the flank appear uniformly dark. In *S. kuehnei* the bars on the caudal peduncle are fused, while in many specimens of *S. aurantiaca* and both *S. hartli* bars in the anterior part of the flank are fused; and in *S. crocotula, S. peninsulae* and *S. robertsi* the partial or complete fusions of bars may be observed on any part of the flank. In *S. ataranensis* the bars are fused and most of the flank is brown, leaving only few interspaces in the middle region of the flank. In *S. tenebrosa* all bars have fused and the flank is plain brown. The extensively broad dark bars interrupted by few interspaces as observed in *S. ataranensis* and some *S. aurantiaca* has erroneously been described as a dark body with light bars.

In the species of the *S. cincticauda* group the interspaces tend to be more colourful than in most other species of *Schistura*. While being light brown or cream white in most *Schistura* species, all or some interspaces are pink, orange or purple in *S. aurantiaca*, some *S. balteata*, *S. crocotula, S. hartli, S. ataranensis* and* S. kuehnei.*

Species of the *S. cincticauda* group have in common a similar body shape with a nearly cylindrical anterior part of the body, a relatively high caudal peduncle with dorsal and ventral crests and short fins with rounded tips. This similar body shape in combination with the small size and often not straight shape of many museum specimens, their different fixation history (ethanol or formalin, concentration, age) made morphometric characters nearly uninformative in the available material (therefore detailed descriptions of the body shape are given in Supplementary Material).

### Characters for species diagnosis

**Figure 6:**
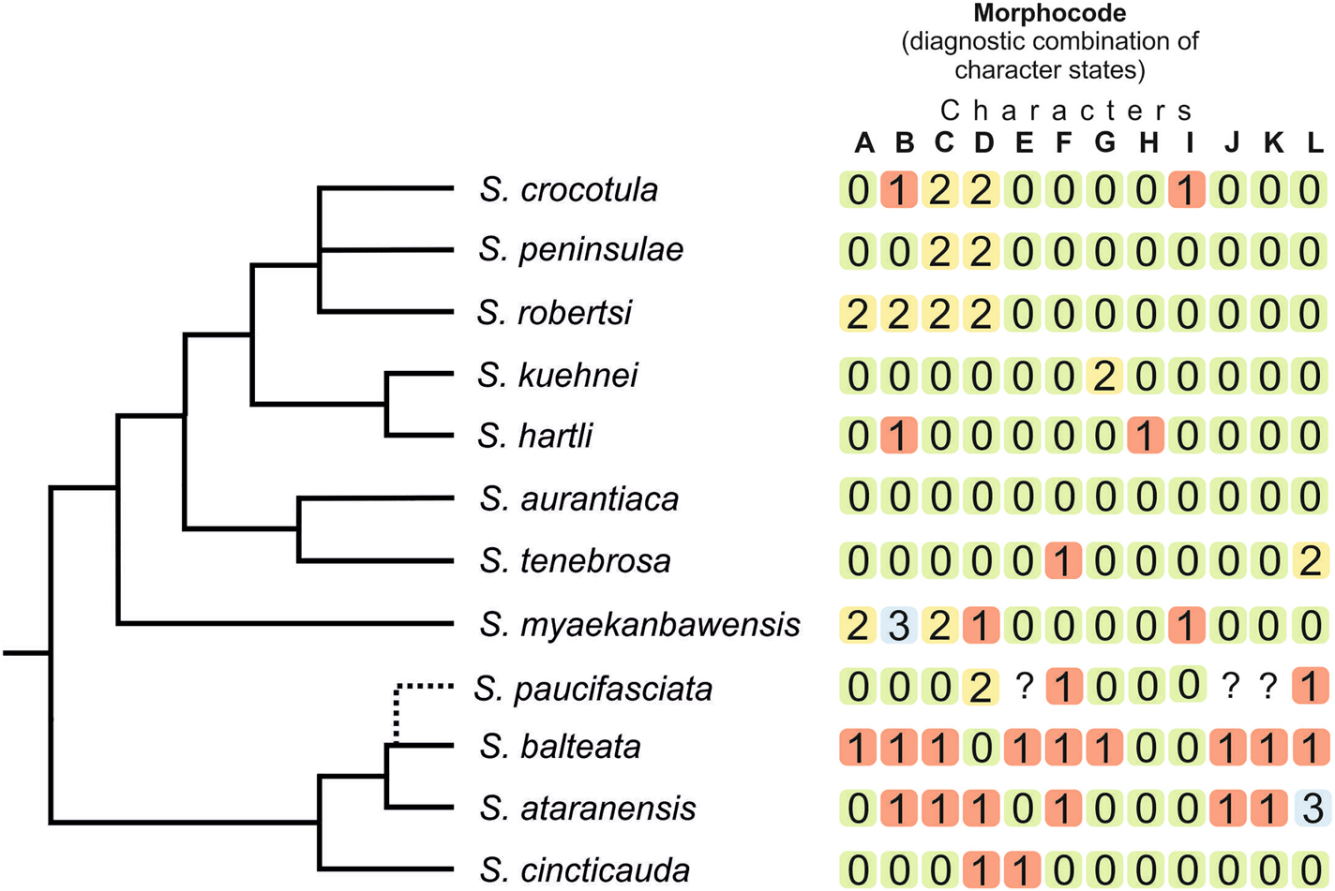
Cladogram based on species tree resulting from multi-species coalescence analyses in *BEAST plus morphocode of the twelve species currently included into the *Schistura cincticauda* species group. The position of *S. paucifasciata* is putative, since no DNA of this species was available. Characters and their states in the morphocode are: (**A**)—number of pectoral-fin rays (0 = 9, 1 = 10–11, 2 = 8); (**B**)—number of branched caudal-fin rays (0 = 9 + 8, 1 = 8 + 8, 2 = 7 + 8, 3 = 8 + 7); (**C**)—length of lateral line (0 = long [ending behind dorsal fin base], 1 = intermediate, 2 = short [ending before middistance from pectoral to pelvic-fin base]); (**D**)—position of anus (0 = half distance between pelvic to anal fin, 1 = closer to pelvic fin, 2 = closer to anal fin); (**E**)—black dots on lower lip (0 = present, 1 = variable); (**F**)—axillary pelvic lobe (0 = absent, 1 = present); (**G**)—black bar on base of caudal fin (0 = present, 1 = absent, 2 = irregular); (**H**)—black blotch posterior of median incision of lower lip (0 = absent, 1 = present) (**I**)—second pair of black dots on lower lip (0 = absent, 1 = present); (**J**)—black dot on base of dorsal fin (0 = present, 1 = absent); (**K**)—black bars on dorsal fin (0 = absent, 1 = present); (**L**)—bars of body (0 = bars numerous, broad or grey, 1 = 2 to 4 very thin black contrasting bars in middle of flank, 2 = all bars fused into plain brown body, 3 = most bars fused leaving few interspaces in middle of flank.

The observed character states that are useful to diagnose species and their expression in each species is given in Table 1 and visualised in Fig. 6. The overview of all variable characters and all species of the *S. cincticauda* species group allows direct one-to-one comparison of the species.

**Table 1:** Comparison of selected morphological characters between the 12 species of the *Schistura cincticauda* species group.

	*S. aurantiaca*	*S. balteata*	*S. cincticauda*	*S. crocotula*	*S. hartli*	*S. ataranensis*	*S. myaekanbawensis*	*S. kuehnei*	*S. robertsi*	S. paucifasciata *	*S. peninsulae*	S. tenebrosa **
n	34	9	2	18	2	11	5	11	46	1	53	36
SL (mm)	21.7–45.4	29.7–57.3	27.9–30.0	17.1–44.8	36.1–41.0	34.9–43.5	36.1–41.0	22.8–29.8	19.5–32.5	46.6	20.5–49.0	32.0–46.0
Anal fin rays	3/5^1^/_2_	3/5^1^/_2_	3/5^1^/_2_	3/5^1^/_2_	3/5^1^/_2_	3/5^1^/_2_	3/5^1^/_2_	3/5^1^/_2_	3/5^1^/_2_	3/5^1^/_2_	3/5^1^/_2_	3/5^1^/_2_
Branched dorsal fin rays	7^1^/_2_	7^1^/_2_	6–7^1^/_2_	7^1^/_2_	7^1^/_2_	7^1^/_2_	7^1^/_2_	7^1^/_2_	7^1^/_2_	7^1^/_2_	7^1^/_2_	7–8^1^/_2_
Pectoral fin rays	9	10–11	9	9	9	9	8	9	7–8	9	9	9–10
Pelvic fin rays	7	7	6–7	7	7	7	7	7	7	7	7	7
Caudal fin rays	/9 + 8/	/8 + 8/	/9 + 8/	/8 + 8/	/8 + 8/	/8 + 8/	/8 + 7/	/9 + 8/	/6–7 + 6–8/	/9 + 8?/	/8–9 + 7–8/	/9 + 7–8/
Lateral line pores	6–26	24–50	15–25	10–18	23–24	18–31	12–18	14–22	7–12	-	9–15	18–30
Supraorb. pores	7	6	5	6	6	6	6	6	6	6	5	6–8
Infraorb. pores	4 + 9	4 + 11	4 + 10	4 + 9	4 + 9	4 + 9	4 + 8–9	4 + 9	4 + 8	4 + 11	4 + 10	4 + 8–11
Supratem. pores	3	3	3	3	3	3	3	3	4	3	3	3
Preoperculo -mandib. pores	9	9	9	9	8	9	9	9	9	9	9	7–10
Black dots on lower lip	Present	Variable	Variable	Present	Present	Present	Present	Present	Present	?	Present	Present
Black bar on caudal-fin base	Light, thin	Absent	Dark, thick	Dark, thick	Dark, thick	Dark, thick	Dark, thick	Incomplete	Dark, thick	Dark, thick	Dark, thick	Dark, thick
Black dot on base of dorsal fin	Present	Absent	Present	Present	Present	Absent	Present	Present	Present	?	Present	Present
Black stripes on dorsal fin	Absent	Present	Absent	Absent	Absent	Present	Absent	Absent	Absent	Absent	Absent	Absent
Position of anus	Middistance pelvic-anal	Middistance pelvic-anal	Closer to pelvic fin	Closer to anal fin	Middistance pelvic-anal	Closer to pelvic fin	Closer to pelvic fin	Middistance pelvic-anal	Closer to anal fin	Closer to anal fin	Closer to anal fin	Middistance pelvic-anal
Length of lateral line ***	Intermediate	Long	Intermediate	Short	Intermediate	Intermediate	Short	Intermediate	Short	Intermediate	Short	Intermediate
Axillary pelvic lobe	Absent	Present	Absent	Absent	Absent	Present	Absent	Absent	Absent	Present	Absent	Present
2nd pair of black dots on lower lip	Absent	Absent	Absent	Present	Absent	Absent	Present	Absent	Absent	?	Absent	Absent
Black blotch on throat	Absent	Absent	Absent	Absent	Present	Absent	Absent	Absent	Absent	?	Absent	Absent

* Data taken from Hora 1929 and Kottelat 1990; ** Data taken from Kangrang et al. 2012; *** “short” = ending before middistance from pectoral to pelvic-fin base, “intermediate” = ending slightly before dorsal-fin origin or under first or second branched ray of dorsal fin, “long” = ending behind dorsal fin base.

### Species overview

#### ***Schistura aurantiaca ***Plongsesthee, Page & Beamish, 2011 (Fig. 4A, Table 1)

**Diagnosis** *Schistura aurantiaca* is distinguished from all species in the *S. cincticauda* species group by the combination of the following character states: axillary pelvic lobe absent; bases of unbranched and first branched dorsal-fin rays with prominent black blotch; broad black bar on base of caudal fin; 9 + 8 branched rays in caudal fin, 9 rays in pectoral fin; colour pattern consisting in 4–12 dark brown bars and 5–11 regular orange interspaces; first interspace slightly before dorsal-fin base; 1–2 interspaces under and one slightly behind dorsal-fin base; sometimes additional 2–5 irregular light orange interspaces on caudal peduncle and 2–4 irregular light orange interspaces between head and dorsal fin; anus midway between posterior extremity of pelvic-fin base and anal-fin origin; incomplete lateral line, reaching to vertical through origin of dorsal fin; colour pattern in smallest examined specimens (20–25 mm SL) already as in adults.

**Distribution** Known from upper Khwae Noi and upper Khwae Yai (Mae Khlong river basin), from upper Moei (Salween river basin), and upper Ataran.

**Remarks **Although formally named only in 2011 by Plongsesthee, Page & Beamish^18^ *S. aurantiaca* had already been described and figured by Rendahl^19^ under the name *S. cincticauda*, on the basis of material from Sukli (16°41′35″ N 98°21′56″ E), a village on the eastern slope of Dawna Hills about 20 km west of Mae Sot (Thailand), in the watershed of Moei River (Salween drainage). The described material can be identified as *S. aurantiaca* by the colour pattern (presence of 10–12 black bars on body in at least small specimens vs. 6–8 bars in *S. cincticauda*). Moreover, the larger specimens in Fig. 18 of Rendahl^19^ show the typical colour pattern of adult *S. aurantiaca*. Hora^17^ also reported *S. cincticauda* from Sukli, but his description and figure do not allow an unambiguous identification.

#### ***Schistura balteata ***(Rendahl, 1948) (Fig. 4B, Table 1)

**Diagnosis** *Schistura balteata* is unique among Nemacheilidae by its adult colour pattern consisting of 2–4 thin black bars below dorsal-fin base and 3–5 saddles in front of dorsal fin; the rest of the body is cream-brown; no black caudal bar. Most similar is *S. paucifasciata*, which shares with *S. balteata* the presence of 2–4 thin black bars on an otherwise light brown body. *Schistura balteata* differs from *S. paucifasciata* by not having a dark bar at base of caudal fin and by presence of 3–5 saddles. Additionally *S. balteata* is characterised by a combination of the following character states: axillary pelvic lobe present; absence of prominent black mark on anterior part of dorsal-fin base; 8 + 8 branched rays in caudal fin, 10–11 rays in pectoral fin; anus midway between posterior extremity of pelvic-fin base and anal-fin origin; incomplete lateral line reaching above anal-fin base.

**Distribution** Known from the type locality (Mahlve Taung mountain range in Tenasserim, Myanmar) and upper Khwae Noi (Mae Klong drainage, Thailand).

#### ***Schistura cincticauda*** (Blyth, 1860) (Fig. 4C, Table 1)

**Diagnosis** *Schistura cincticauda* is distinguished from all other species in the *S. cincticauda* species group by the combination of the following character states: axillary pelvic lobe absent; base of unbranched and first branched dorsal-fin rays with prominent black blotch; complete and broad black bar on base of caudal fin; 9 + 8 branched rays in caudal fin, 9 rays in pectoral fin; colour pattern consisting of 6–8 regular brown bars and regular yellow interspaces as wide as bars; anus closer to posterior extremity of pelvic-fin base than to anal-fin origin; incomplete lateral line, ending before vertical through origin of dorsal fin.

**Distribution** Known only from Moei River watershed (Salween drainage).

**Remarks ***Schistura cincticauda* was originally described from 'Tenasserim Provinces'^20^, at that time including Myanmar between 11° and 17°N. Kottelat^11^ redescribed *S. cincticauda* and designated a neotype, which fixes the type locality in the Mae Nam Moei watershed, about 30 km North of Mae Sot. The Mae Nam Moei makes the border between Thailand and the earlier 'Tenasserim Provinces' of Myanmar. For confusion with *S. aurantiaca*, see above.

#### ***Schistura crocotula ***Plongsesthee, Kottelat & Beamish, 2013 (Fig. 4D*, *Table 1)

**Diagnosis** *Schistura crocotula* is distinguished from all other species in the *S. cincticauda* species group by the combination of the following character states: axillary pelvic lobe absent; base of unbranched and first branched dorsal-fin rays with prominent black blotch; complete and broad black bar on base of caudal fin; 8 + 8 branched rays in caudal fin, 9 rays in pectoral fin; colour pattern consists of dark brown bars and prominent regular orange to red interspaces; anus closer to anal-fin origin than to posterior extremity of pelvic-fin base; incomplete lateral line, reaching at most to vertical through middistance between pectoral-fin base and origin of dorsal fin.

**Distribution** Observed in Bang Saphan and Pranburi rivers (Prachuap Khiri Khan Province, Thailand) and Lenya River (Tanintharyi Region, Myanmar).

#### ***Schistura paucifasciata*** (Hora, 1929) (Fig. 4E*, *Table 1)

**Diagnosis** *Schistura paucifasciata* is identified as member of the *S. cincticauda* group by having an incomplete lateral line, a small body size (max 54.3 mm TL according to Hora^17^) and an emarginate caudal fin. Most likely related to *S. balteata*, with which it shares a rare colour pattern (2–4 thin dark bars below dorsal fin, body cream-brown), distinguishing these two species from all other Nemacheilidae*.* It is distinguished from *S. balteata* by the absence of saddles on the back and by presence of a broad black bar on caudal-fin base. *Schistura paucifasciata* is further characterised by the combination of the following character states: axillary pelvic lobe present; absence of prominent black mark on anterior part of dorsal-fin base; complete and broad black bar on base of caudal fin; 10–11 rays in pectoral fin; anus closer to anal-fin origin than to posterior extremity of pelvic-fin base; incomplete lateral line, ending just before dorsal-fin origin.

**Distribution** Known only from the type locality in the middle Irrawaddy basin, Kyaukme district, north-western Shan State, Myanmar.

**Remarks ***Schistura paucifasciata* has not been collected again since 1911^17^. Menon^21^ considered that *S. paucifasciata* is a synonym of *S. cincticauda* after having examined the holotype of *S. paucifasciata*; he did not examine material of *S. cincticauda*. His description of *S. cincticauda* in fact is that of the holotype of *S. paucifasciata*.

The BMNH paratype has the lower lip pushed down in a non-natural way and it is impossible to observe the black marks on the lower lip. In *S*. *balteata*, which shares the pattern of a few blackish bars below the dorsal fin, some specimens do not have black marks on the lower lip; possibly, their apparent absence in the only available specimen of *S. paucifasciata* has no significance and at this stage should not be retained as diagnostic for the species. The holotype and paratypes housed by the Zoological Survey of India could not be investigated.

Having three black bars below the dorsal fin on an otherwise uniformly brown body is a character shared with *S. balteata*. The bars are more widely spaced in *S. paucifasciata* than in *S. balteata*. The colour pattern of juvenile *S. paucifasciata*, as illustrated by Hora^17^ has some similarities with that of *S. aurantiaca*, with 8 grey bars separated by whitish narrow interspaces. With increasing size, the bars fade away, the body becomes uniform reddish brown. At the same time, 3 or 4 narrows bars appear in the interspaces below the dorsal fin. Hora described them as deep reddish brown.

#### ***Schistura robertsi ***Kottelat, 1990 (Fig. 4F*, *Table 1)

**Diagnosis** *Schistura robertsi* is distinguished from all other species in the *S. cincticauda* species group by the combination of the following character states: axillary pelvic lobe absent; base of unbranched and first branched dorsal-fin rays with prominent black blotch; complete and broad black bar on base of caudal fin; 6–7 + 7–8 branched rays in caudal fin; 7–8 rays in pectoral fin; colour pattern consisting of 1–13 regular brown bars and 1–12 regular yellow interspaces as wide as bars; number of bars and their width very variable between and also within populations; anus closer to anal-fin origin than to posterior extremity of pelvic-fin base; incomplete lateral line, reaching at most to vertical through halfway between pectoral-fin base and origin of dorsal fin.

**Figure 7:**
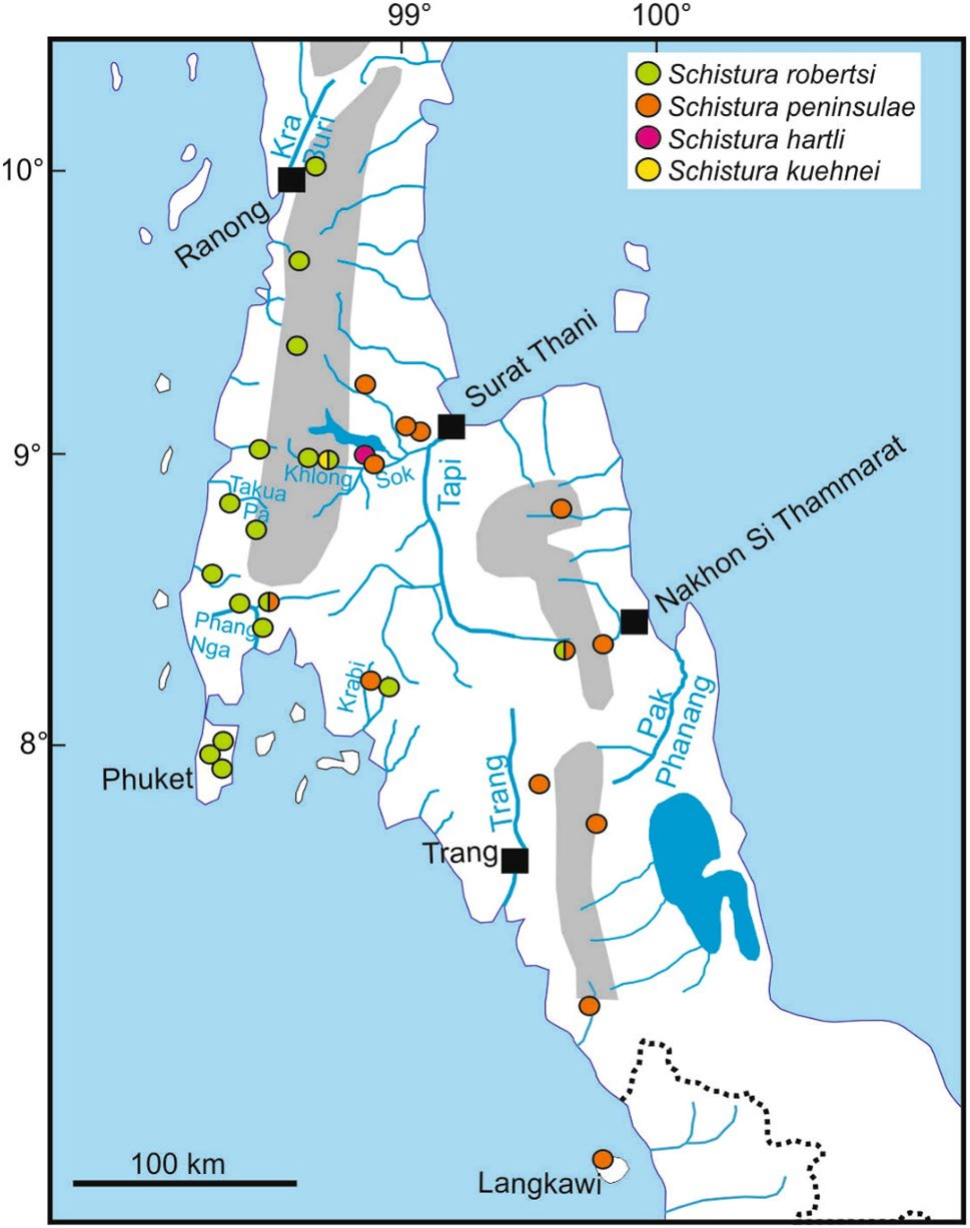
Detailed geographic origin of samples of the southernmost species of the *Schistura cincticauda* species group.

**Distribution** Widely distributed in most drainages of the Andaman Sea slope of Thailand, from the Isthmus of Kra southwards to Phuket Island. It was also found in some localities east of the Tenasserim Range in the upper Khlong Sok and upper Tapi River, which drain to the Gulf of Thailand, and in the Krabi River basin (Fig. 7). It occurs in sympatry with *S. kuehnei* in the upper Khlong Sok and with *S. peninsulae* in the upper Tapi.

**Remarks **Kottelat^11^ considered *S. robertsi* to occur on the Malay Peninsula from Ranong to Songkhla. Meanwhile genetic data have demonstrated that *S. robertsi* sensu Kottelat^11^ consists of two lineages, one in the Andaman Sea drainage from Phang Nga to Ranong provinces (Thailand) and one from Phang Nga and Surat Thani provinces in the north to northern Malaysia in the south, including Langkawi Islands (Fig. 1). Kottelat's original description of *S. robertsi* was mostly based on paratypes now identified as *S. peninsulae*. The fin-ray counts of the holotype were not mentioned separately and the diagnosis is misleading. The holotype was re-examined for the present study. It has 7 + 7 branched caudal-fin rays, 8 and 7 pectoral-fin rays (right and left, respectively). The holotype of *S. robertsi* has all the characters here considered diagnostic for the northern species and the type locality is within that range. *Schistura robertsi* is unique among the species of the *S. cincticauda* species group by having often fewer branched rays in the upper lobe (6–7) of the caudal fin than in the lower lobe (7–8).

#### ***Schistura tenebrosa ***Kangrang, Page, Beamish, 2012 (Fig. 4G*, *Table 1)

**Diagnosis** *Schistura tenebrosa* is distinguished from all other species in the *S. cincticauda* species group by the combination of the following character states: body plain brown without any marks; axillary pelvic lobe present; base of unbranched and first branched dorsal-fin rays with prominent black blotch; presence of black bar on base of caudal fin; 9 + 8 branched rays in caudal fin, 9–10 rays in pectoral fin; anus midway between posterior extremity of pelvic-fin base and anal-fin origin; lateral line incomplete, ending just before vertical through dorsal-fin origin.

**Distribution** Known only from two small streams draining into Vajiralongkorn reservoir, Mae Kwai Noi, Mae Khlong drainage, Kanchanaburi province, Thailand.

**Remarks **Material of this species was not observed personally, the data were obtained from the original description and from personal communication with the second author of the original description. The general body shape of the type material of *S. tenebrosa* was very similar to the species of the *S. cincticauda* group. A photo of the mouth of the holotype shows the presence of prominent black marks in the lower lip as diagnostic for the *S. cincticauda* species group. Cytochrome b data indicate *S. tenebrosa* as member of the *S. cincticauda* species group (position indicated in Fig. 1).

#### ***Schistura ataranensis***, new species (Fig. 5A, Tables 1 and 2; Supplementary Material Figs. S1, S2)

**Table 2:** Morphometric values of *Schistura ataranensis, S. hartli, S. myaekanbawensis, S. kuehnei* and *S. peninsulae.*

	*S. ataranensis*		*S. hartli*		*S. myaekanbawensis*		*S. kuehnei*		*S. peninsulae*	
	Holotype	Range	Holotype	Paratype	Holotype	Range	Holotype	Range	Holotype	Range
Standard length	43.8	36.0–43.8	36.6	41.0	29.8	23.2–29.8	37.1	31.6–37.1	36.8	32.4–39.4
In percent of standard length										
Total length	121.5	119.5–127.8	123.2	123.5	125.5	124.4–126.3	125.1	123.6–125.1	124.5	122.9–128.0
Dorsal head length	17.4	17.4–23.7	20.5	19.1	18.1	18.1–21.1	22.9	22.2–23.3	19.0	18.6–21.6
Lateral head length	20.1	20.1–25.1	23.2	21.3	21.8	21.8–25.0	24.5	23.7–26.1	22.8	22.0–24.8
Predorsal length	52.3	52.2–57.3	52.7	55.7	54.7	54.7–56.9	56.1	54.4–57.5	56.0	54.5–57.0
Pre-pelvic length	53.0	53.0–58.0	53.7	51.1	53.0	52.3–54.3	54.4	53.2–55.1	49.7	49.6–55.1
Pre-anus length	65.5	65.5–68.1	69.8	63.7	64.8	64.8–65.4	67.7	67.0–69.7	70.4	67.3–71.0
Preanal length	79.5	76.7–82.3	81.0	77.3	78.5	78.5–81.0	78.7	77.8–81.2	78.0	76.1–79.8
Head depth at eye	11.0	10.9–13.3	10.5	9.8	9.1	9.1–10.8	12.7	12.7–13.8	10.6	10.1–14.2
Head depth at nape	14.6	12.8–14.8	13.2	12.8	11.4	11.4–13.4	13.5	13.5–14.9	13.0	12.7–16.0
Body depth	14.8	14.8–17.1	17.6	14.8	13.8	13.8–15.8	16.2	16.2–17.1	17.7	14.9–21.0
Depth of caudal peduncle	12.1	12.1–15.6	13.2	12.8	13.1	13.1–15.1	13.7	13.7–14.9	15.2	11.8–15.2
Length of caudal peduncle	11.9	11.6–16.0	12.0	13.7	16.1	15.9–18.1	13.7	13.7–15.5	14.4	14.4–18.4
Snout length	8.9	8.9–15.3	9.5	9.3	12.1	11.6–12.9	15.1	15.1–15.8	9.0	9.0–11.3
Head width at nares	10.3	10.3–13.1	11.0	10.4	10.4	9.8–11.5	15.6	14.5–15.6	11.7	10.7–16.5
Maximum head width	14.6	14.6–18.0	16.1	15.8	14.1	14.1–15.9	18.9	18.9–19.4	17.1	15.6–19.3
Body width at dorsal-fin origin	14.8	10.9–14.8	11.2	10.7	10.7	10.7–12.2	13.5	13.5–14.6	13.9	11.3–17.0
Body width at anal-fin origin	8.9	6.5–9.3	6.3	6.3	7.7	7.3–8.2	11.1	9.8–11.1	9.8	8.2–10.9
Eye diameter	4.8	4.1–5.5	2.9	3.3	3.0	3.0–3.4	4.3	4.3–4.7	3.3	3.1–4.2
Interorbital distance	5.9	5.9–7.8	6.6	6.6	7.4	6.9–7.5	8.4	8.0–8.4	9.2	6.5–9.2
Length of upper caudal lobe	20.5	20.2–22.9	22.7	23.0	21.5	21.5–23.7	22.6	20.9–22.6	0.0	20.5–23.5
Length of lower caudal lobe	20.5	19.7–21.8	22.0	22.1	20.5	19.4–22.8	21.8	20.6–21.8	22.0	19.4–22.5
Length of medium caudal rays	16.2	16.2–18.1	19.8	20.8	17.8	15.8–19.4	20.2	18.9–20.2	20.1	17.7–20.8
Depth of anal fin	15.8	15.8–18.8	18.5	18.6	17.1	15.6–17.7	18.1	18.1–18.6	17.7	15.7–19.7
Length of pelvic fin	15.3	15.3–19.3	15.9	18.0	17.4	16.9–19.0	17.3	17.3–18.7	17.9	17.0–19.4
Length of pectoral fin	18.0	18.0–22.0	19.0	21.3	19.5	19.5–20.3	17.5	17.5–19.7	17.4	17.5–20.3
In percent of dorsal head length										
Snout length	51	51–68	46	49	67	55–68	66	66–71	47	47–59
Eye diameter	27	20–28	14	17	17	16–17	19	19–21	17	15–20
Interorbital distance	34	30–37	32	34	41	33–41	37	34–37	49	32–49

**Holotype** ZRC 61579, 43.8 mm SL; Myanmar: Kayin Province: upper Ataran basin (Andaman Sea basin), collected for ornamental fish trade, no details. **Paratypes** IAPG A11005, 1, 35.6 mm SL; ZRC 61580, 1, 41.5 mm SL; CMK 28809, 2, 37.0–41.0 mm SL; collected with holotype.

**Diagnosis** *Schistura ataranensis* is distinguished from all other species in the *S. cincticauda* species group by having 2–3 black stripes on the dorsal fin. The following combination of character also distinguishes the species: absence of a prominent black mark on the anterior part of dorsal-fin base; axillary pelvic lobe present; complete and broad black bar at base of caudal fin; 8 + 8 branched rays in caudal fin, 9 rays in pectoral fin; colour pattern always includes two prominent regular paler interspaces (orange to pink in life), one below dorsal-fin origin, one before; 1–2 additional, irregular small interspaces on back in front of dorsal and up to 4 irregular yellow to orange interspaces between dorsal- and caudal-fin bases; anus closer to posterior extremity of pelvic-fin base than to anal-fin origin; lateral line incomplete, ending under anterior half of dorsal-fin base.

**Colour pattern** In preserved specimens, body (except belly) variable, but mainly plain brown, with a few vertically elongated pale marks (plain brown areas in fact homologous to fused bars in other species; pale marks homologous to interspaces). Two light interspaces, one below dorsal-fin base and the other in front of dorsal-fin origin, orange to pink in life (light yellow after fixation), continuous with their contralaterals across back. Bar at base of caudal fin broad, black, complete, extending across ventral and dorsal crests, forming a complete ring around end of caudal peduncle. Dorsal fin with 2 or 3 black stripes made of pigments on rays and membranes. Caudal-fin rays (and in most specimen also each pectoral-, pelvic- and anal-fin ray) with dark pigment along whole length of ray. Small dark spots present on each side of median interruption of lower lip. Head entirely dark brown to dark grey, with black patches around eyes and nostrils, and a dark stripe from front of eyes to snout. Rostral barbels pigmented, outer one entirely black and inner one with dark base and light tip. Maxillary barbel unpigmented.

**Distribution** Known only from the upper Ataran River drainage, in Myanmar.

**Etymology** Named after River Ataran, where the type series was collected. An adjective, *-is*, *-is*, *-e*.

**Remarks ***Schistura ataranensis* is traded as ornamental fish since at least 2006 under names like ‘Sumo loach’, ‘Sumo loach II’, ‘*Schistura* cf. *balteata*’^22^. However, pictures of fishes from the ornamental fish trade suggest that additional species are confused under these names.

#### ***Schistura hartli***, new species (Fig. 5B, Tables 1 and 2; Supplementary Material Figs. S3, S4)

**Holotype** ZRC 61581, 41.0 mm SL; Thailand: Surat Thani Province: small stream in Khao Sok NP, around 8°57′N 98°36′ E; A. Hartl. **Paratype** CMK 28810, 1, 36.6 mm SL; collected with holotype.

**Diagnosis** *Schistura hartli* is distinguished from all other species in the *S. cincticauda* species group by the combination of the following character states: absence of axillary pelvic lobe; presence of a black patch on throat, behind median interruption of lower lip; base of unbranched and first branched dorsal-fin rays with prominent black mark; black bar at base of caudal fin; 8 + 8 branched rays in caudal fin, 9 rays in pectoral fin; flank with 7–10 bars and 8–11 broad pink interspaces; anus more or less midway between posterior extremity of pelvic-fin base and anal-fin origin; lateral line incomplete, ending before vertical through dorsal-fin origin.

**Colour pattern** Body with 7–10 broad dark brown bars with broad pink interspaces (turn light yellow after fixation in formalin or ethanol), all fused with their contralateral on dorsal midline. Bars reaching ventrally to about level of pectoral-fin base. First interspace behind neck yellow in life. Black bar at base of caudal fin complete. Dorsal-fin with circular to squarish black spot on base of unbranched and anterior 1–2 branched rays. Dorsal and caudal fins with dark pigments along rays, intensity decreasing with distance from base. Lower lip with a small dark spot on each side of median interruption; a black patch on throat, behind median interruption of lower lip. Head dark brown to nearly black, with light brown blotches on gill cover. Maxillary barbel with small patches of pigments at base. Rostral barbels pigmented, outer one entirely black, inner one with dark base and light tip.

**Distribution** Known only from three specimens, collected in Khao Sok NP in a small forest stream (Fig. 7). Exact position of the locality unknown.

**Etymology** Named for Andreas Hartl, who collected the type material. Noun in genitive, indeclinable.

#### ***Schistura myaekanbawensis***, new species (Fig. 5C, Tables 1 and 2; Supplementary Material Fig. S5)

**Holotype** MHNG 2790.081, 29.8 mm SL; Myanmar: Tanintharyi Region: Tanintharyi drainage: Kami Chaung, 14°20′52″ N 98°31′28″ E, 255 masl; M. Kottelat et al., 2 May 2014. **Paratypes** CMK 24993, 4 (one in ethanol), 22.8–29.4 mm SL; collected with holotype.—CMK 24971, 3, 22.7–27.3 mm SL; ZRC 64851, 2, 26.6-28.1 mm SL; Myanmar: Tanintharyi Region: Tanintharyi drainage: Kami Chaung, 14°20′07″ N 98°31′04″ E, 244 masl; M. Kottelat et al., 2 May 2014.﻿

**Diagnosis** *Schistura myaekanbawensis* is distinguished from all other species in the *S. cincticauda* species group by the combination of the following character states: presence of a second black mark on lower lip close to corner of mouth; an elongate swelling along ventral midline between anus and anal-fin origin; absence of axillary pelvic lobe; base of unbranched and first branched dorsal-fin rays with prominent black blotch; black bar at base of caudal fin; 8 + 7 branched rays in caudal fin; 8 rays in pectoral fin; anus closer to posterior extremity of pelvic-fin base than to anal-fin origin; lateral line incomplete, reaching at most to 2/3 of distance between pectoral-fin base and vertical through dorsal-fin origin.

**Colour pattern** Body pale brown to yellowish, paler on belly. Flank with 6–12 dark brown bars, separated by interspaces narrower than bars. Bars meeting contralaterals on dorsal midline. Bars in front of anal fin reaching downwards about to level of pectoral fin, those on caudal peduncle reaching contralaterals on ventral midline. Interspaces between anterior most bars indistinct or only weakly contrasted (more contrasted in smallest specimens). Anterior interspace through neck and posterior margin of opercle to pectoral-fin base, thin and incomplete. Dark bar at base of caudal fin complete and broad, widest on adipose crests. Dorsal-fin with triangular to squarish black spot on base of unbranched and anterior 1–2 branched rays. All fins with dark pigment on rays, near branching points. A small black spot on each side of median interruption on lower lip (usually poorly contrasted); another, larger black mark on each corner of mouth (Fig. 2). Head dark brown with darker stripe from snout to eye. Upper lip dark brown, lower one light brown. Maxillary barbel with small patches of black pigments, outer rostral barbel black, inner rostral barbel with black base and light tip.

**Distribution** Only known from Kami Chaung, a shallow stream with moderate flow in the upper Tanintharyi drainage. The individuals were collected in riffles, with a substrate of gravel to small stones.

**Etymology** Named after the Myaekanbaw region, where the type series was collected. An adjective, *-is*, *-is*, *-e*.

**Remarks ***Schistura myaekanbawensis* is unique among the species of the *S. cincticauda* group by the presence of a narrow elongated swelling along the vertical midline between the anus and the anal-fin origin. *Schistura myaekanbawensis* also has bars and interspaces narrower than the other species of this group. The presence of the swelling maybe seasonal.

#### ***Schistura kuehnei***, new species (Fig. 5D, Tables 1 and 2; Supplementary Material Figs. S6, S7)

**Holotype** ZRC 61582, 37.1 mm SL; Thailand: Surat Thani Province: Tapi River drainage: Khlong Sok watershed: small forest stream shortly before entering Khlong Sok, about 8 54′48″ N 98°31′30″ E; J. Kuehne. **Paratypes** ZRC 61583, 1, 31.6 mm SL; CMK 28811, 2, 33.9–35.6 mm SL; collected with holotype.

**Diagnosis** *Schistura kuehnei* is distinguished from all other species in the *S. cincticauda* species group by the combination of the following character states: absence of axillary pelvic lobe; base of unbranched and first branched dorsal-fin rays with prominent black blotch; incomplete black bar at base of caudal fin; 9 + 8 branched rays in caudal fin, 9 rays in pectoral fin; dark brown body with 7–10 bars and narrow pink interspaces; bars fused in posterior third of body, without pale interspaces; anus about midway between posterior extremity of pelvic-fin base and anal-fin origin; lateral line incomplete, reaching at most to vertical through dorsal-fin origin.

**Colour pattern** Body pale brown, paler on belly, with 7–10 broad brown bars separated by thinner pink interspaces (light yellow after fixation). Bars and interspaces sometimes continuous with contralaterals across back. In some specimens, interspaces just before and below dorsal-fin base broader than others. Bars and interspaces more sharply contrasted between head and dorsal fin; from there gradually less contrasted, then body uniformly brown from anal-fin origin. Dorsal-fin with triangular to circular black spot on base of unbranched and anterior 1–2 branched rays. Rays of dorsal and caudal fin covered by dark pigments along whole length, denser and somewhat extending on membranes near first branching points of rays. Dark bar at caudal fin base incomplete, thin and with a small interruption at upper third, not reaching dorsal and ventral crest. A prominent and large black mark on each side of median interruption on lower lip, stretching over one third of length of lower lip. Head dark brown, with dark stripe from front of eyes to snout, small dark patches between eyes and nostrils; nostril flap dark; interorbital space, tip of snout and sides of head light brown. Maxillary barbel without pigments. Outer rostral barbel completely black, inner one with dark base and light tip.

**Distribution** Known only from a small forest stream draining from Khao Sok NP into stream Khlong Sok (River Tapi drainage) in Surat Thani province (Fig. 7); at the type locality it co-occurs with *S. robertsi*.

**Etymology** Named after Jens Kühne, in acknowledgement of his long-time support of our ichthyological work in Southeast Asia. Noun in genitive, indeclinable.

#### ***Schistura peninsulae,*** new species (Fig. 5E, Tables 1 and 2; Supplementary Material Fig. S8)

**Holotype** ZRC 61584, 35.6 mm SL; Malaysia: Kedah province: Langkawi Island: waterfall below seven wells (Sungai Borau drainage), 6°23′ N 99°40′ E. **Paratypes** CMK 29091, 1, 38.2 mm SL; collected with holotype.—ZRC 61585, 5, 32.5–38.4 mm SL; Thailand: Nakhon Si Thammarat Province: Amphoe Chang Klang: Tapi drainage: waterfall 'Khao Mhen' 8°18′ N 99°39′ E. J. Kühne.

**Diagnosis** *Schistura peninsulae* is distinguished from all other species in the *S. cincticauda* species group by the combination of the following character states: absence of axillary pelvic lobe; base of unbranched and first branched dorsal-fin rays with prominent black blotch; black bar at base of caudal fin; 8–9 + 7-8 branched rays in caudal fin; 9–10 rays in pectoral fin; anus closer to anal-fin origin than to posterior extremity of pelvic-fin base; lateral line incomplete, reaching at most to vertical through halfway between pectoral-fin base and origin of dorsal fin.

**Colour pattern** Body background yellowish to pale brown, with 2–10 dark brown bars separated by interspaces narrower than bars. Width, number and shape of bars variable; some bars branched near inferior extremity. Bars and interspaces usually sharply contrasted, but in some specimens bars and interspaces not contrasted and body uniform brown, with only two interspaces remaining, one behind neck and one in front of caudal-fin base. First interspace directly behind neck, reaching ventrally to belly, more contrasted than other interspaces. Black bar at base of caudal fin complete and wide. Dorsal fin with a black mark on base of unbranched and first two branched dorsal-fin rays. On dorsal and caudal fins, dark pigments along each ray. A prominent and large black mark on each side of median interruption of lower lip, stretching over one third of length of lip. Top and sides of head dark brown, ventral side light yellow. A black stripe between eyes and snout, nasal tube dark brown, interorbital space and tip of snout light brown. Maxillary barbel with small patches of black pigments; outer rostral barbel black; inner rostral barbel with dark base and light tip.

**Distribution** Widely distributed on the Malay Peninsula, from Phang Nga (Thailand) to Langkawi (Malaysia) on the west slope and from northern Surat Thani province to Phatthalung province (Thailand) on the east slope (Fig. 7). *Schistura peninsulae* occurs in syntopy with *S. robertsi* in at least one locality in the uppermost Tapi river basin, in a small stream in the Phang Nga river basin and potentially in some localities on Phuket Island (see Remarks, below).

**Etymology** Named after its wide distribution on the Malay Peninsula; it also is the most southern species of *Schistura* on the Malay Peninsula. A noun in genitive, indeclinable.

**Remarks ***Schistura peninsulae* and *S. robertsi* have similar body shape and colour pattern. The clear genetic distance and the case of syntopy without hybridisation in the upper River Tapi basin show that they are distinct evolutionary units. The only external characters that allow to distinguish them are the numbers of rays in pectoral fin (9–10 in *S. peninsulae* vs 7–8 in *S. robertsi*) and branched rays in caudal fin (8–9 + 7–8 in *S. peninsulae* vs 6–7 + 7–8 in *S. robertsi*).

The identity of the specimens on Phuket Island is not yet clear. It is possible that *S. robertsi* and *S. peninsulae* co-occur, or that only *S. robertsi* is present, but with a wider range in the number of branched caudal-fin rays. The only specimen from Phuket that was analysed genetically was identified as *S. robertsi* in nuclear and mitochondrial genes as well as by morphology (8 pectoral-fin rays, 7 + 8 branched caudal-fin rays). All 20 formalin-fixed specimens from 4 Phuket lots housed in ZRC (ZRC 43693, ZRC 45718, ZRC 49182, ZRC 60821) have 8 pectoral-fin rays (diagnostic for *S. robertsi*), but 8 specimens have 7 + 7 or 7 + 8 branched caudal-fin rays (diagnostic for *S. robertsi*), while 12 specimens have 8 + 8 branched caudal-fin rays (diagnostic for *S. peninsulae*). In 8 paratypes of *S. robertsi* from Phuket (MCZ 49164) examined in 1988^11^, 4 have 7 + 8 branched caudal-fin rays (diagnostic for *S. robertsi*) and 4 have 8 + 8 (diagnostic for *S. peninsulae*).

### Key to species of the *S. cincticauda* species group

- Axillary pelvic lobe present. …………………………………………………..….….. 2
  - No axillary pelvic lobe. …..………………………………………………..……....... 5
- Colour pattern consisting of 2–4 thin dark bars below dorsal fin. ……………………… 3
  - Other colour pattern. …………...........……………..……………………………….. 4
- No black bar at base of caudal fin; 3–5 black saddles on back; 8 + 8 branched caudal-fin rays. ..…………………………….………..………..……………………. *S. balteata*
  - A black bar at base of caudal fin; no dorsal saddles; 9+8 branched caudal-fin rays. …………................……………………………………………………* S. paucifasciata*
- Dorsal fin with 2–3 black stripes; 8 + 8 branched caudal-fin rays; no black mark on anterior part of dorsal-fin base……….……….…………………..…….. *S. ataranensis*
  - Dorsal fin with a single row of spots or plain; 9+8 branched caudal-fin rays; a prominent black mark on anterior part of dorsal-fin base ………………….* S. tenebrosa*
- Anus closer to pelvic-fin base than to anal-fin origin. …………….………..………. 6
  - Anus closer to anal-fin origin than to pelvic-fin base, or at equal distance. …..….… 7
- 8 pectoral-fin rays; 8 + 7 branched caudal-fin rays; a second pair of black dots on lower lip close to corners of mouth. …………………….…….………. *S. myaekanbawensis*
  - 9-11 pectoral-fin rays; 9+8 branched caudal-fin rays; a single pair of black dots on lower lip ..................………... ……………….......................................... *S. cincticauda*
- Anus at equal distance between pelvic-fin base and anal-fin origin. ………………… 8
  - Anus closer to anal-fin origin than to pelvic-fin base. …………………....….…...… 9
- 8 + 8 branched caudal-fin rays; black bar at caudal-fin base regular (without interruption); a black patch on throat, posteriorly of median interruption of lower lip ……………..………………………………………………………………………………………….. *S. hartli*
  - 9+8 branched caudal-fin rays; black bar at base of caudal-fin irregular (with interruption); no black patch on throat ................……...………………........ *S. kuehnei*
- 6–7 branched rays in upper caudal-fin lobe; 7–8 rays in pectoral fin. …..… *S. robertsi*
  - 8-9 branched rays in upper caudal-fin lobe; 9-11 rays in pectoral fin. …....….…… 10
- Brown bars with yellowish interspaces in live specimens; all interspaces very narrow (at most 1/3 of width of bars) and all of similar width. …….…….………. *S. peninsulae*
  - Brown bars with orange to red interspaces on live specimens; interspaces wider than 1/2 of width of bars; interspaces under dorsal fin wider. ………............................…. 11
- 8 + 8 branched caudal-fin rays; lateral line reaching at most halfway between pectoral-fin base and dorsal fin origin; coastal streams in Prachuap Khiri Khan Province, southern Thailand. ..… …………………………………………………..…..…… *S. crocotula*
  - 9+8 branched caudal-fin rays; lateral line reaching vertical through dorsal-fin origin; head waters of Mae Klong, Ataran and Moei rivers. ………..…..…....… *S. aurantiaca*

### Supplementary Information

Supplementary Information.

## Data Availability

Details on morphological data are given in the Supplementary Material and on request from the authors. Sequence data are available from GenBank (Accession numbers: MK886860-MK887044). Specimens housed in public collections and CMK can be investigated according to the collection’s regulations.
