# Supplementary material for: Revision of the Schistura cincticauda species group (Teleostei, Nemacheilidae) using molecular and morphological markers
Source: Sci Rep. 2023 Oct 9;13:16996. doi: 10.1038/s41598-023-42852-1 (PMC10562404; doi:10.1038/s41598-023-42852-1)
Supplement: Supplementary file 1 — Supplementary Information. [file 41598_2023_42852_MOESM1_ESM.doc]

**Revision of the *Schistura cincticauda* species group (Teleostei, Nemacheilidae) using molecular and morphological markers**

Tomáš Dvořák, Jörg Bohlen, Maurice Kottelat, Vendula Šlechtová

**SUPPLEMENTARY MATERIAL**

**Part A) Material analysed**

**Morphological material lists:** Voucher code, number of specimens, standard length of specimens, country, province, first order river drainage, second order river drainage

**Genetic material lists:** Voucher code, name of first gene, first GenBank accession number, second gene, second GenBank accession number

**Collection abbreviations:** CMK, Collection of Maurice Kottelat, Delémont, Switzerland; IAPG, Collection of the Institute of Animal Physiology and Genetics, Liběchov, Czech Republic; MCZ, Museum of Comparative Zoology, Harward University, Cambridge, USA; MHNG, Muséum d'Histoire Naturelle, Geneva, Switzerland; NHM, Natural History Museum, London, United Kingdom; UF, University of Florida, Gainsville, USA; ZRC Zoological Reference Collection, Lee Kong Chian Natural History Museum, National University of Singapore, Singapore.

***Schistura aurantiaca* Plongsesthee, Page & Beamish, 2011**

**Morphological material:** IAPG A0954 – A0957, 4, 38.4–43.7 mm SL; Myanmar: Mon State, Ataran, no details. — IAPG A4417 – A4421, 5, 24.1–35.3 mm SL; Thailand: Kanchanaburi Province, Mae Klong, Khwae Noi. — IAPG A9557-A9559, A9580, 4, 33–40.4 mm SL; Thailand: Tak Province, Mae Klong, Khwae Yai. — IAPG A9584, 1, 28.9 mm SL; Thailand: Tak Province, Salween, Moei. — IAPG A10902, A11024 – A11029, A11067, 9, 24.7–41.5 mm SL; Thailand: Tak Province, Mae Klong, Mae Klong Noi. — IAPG A10979 – A11001, A11265 – A11274, 33, 26.3–44.7 mm SL; Thailand: Tak Province, Mae Klong, Mae Klong Noi. — IAPG A11011 – A11016, 5, 21.5–34.9 mm SL; Thailand: Tak Province, Salween, Gyaing.

**Genetic material (Voucher code plus GenBank accession numbers):** IAPG A0954: cyt b MK886950, IRBP MK886861; IAPG A0955: cyt b MK886951, IRBP MK886862; IAPG A0956: cyt b MK886952, IRBP MK886863; IAPG A0957: cyt b MK886953, IRBP MK886864; IAPG A4417: cyt b MK886987, IRBP MK886896; IAPG A4418: cyt b MK886988, IRBP MK886897; IAPG A4419: cyt b MK886989, IRBP MK886898; IAPG A4420: cyt b MK886990, IRBP MK886899; IAPG A9580: cyt b MK887018, IRBP MK886926; IAPG A9584: cyt b MK887019, IRBP MK886927; IAPG A10902: cyt b MK887025, IRBP MK886933; A10979: cyt b MK887028, IRBP MK886936; A11000: cyt b MK887029, IRBP MK886937; A11010: cyt b MK887032, IRBP MK886940; A11067: cyt b MK887038, IRBP MK886946.

***Schistura balteata* (Rendahl, 1948)**

**Morphological material:** IAPG A2554 – A2557, 4, 30.1–32.5 mm SL; Myanmar: Mon State, no details. — IAPG A4413 – A4416, 11754-11755, 6, 25.7–44.7 mm SL; Thailand: Kanchanaburi province, Mae Klong, Khwae Noi.

**Genetic material (Voucher code plus GenBank accession numbers):**

IAPG A0324: cyt b MK886949, IRBP MK886860; IAPG A2554: cyt b MK886971, IRBP MK886880; IAPG A2555: cyt b MK886972, IRBP MK886881; IAPG A2556: cyt b MK886973, IRBP MK886882; IAPG A2557: cyt b MK886974, IRBP MK886883; IAPG A4413: cyt b MK886983, IRBP MK886892; IAPG A4414: cyt b MK886984, IRBP MK886893; IAPG A4415: cyt b MK886985, IRBP MK886894; IAPG A4416: cyt b MK886986, IRBP MK886895.

***Schistura cincticauda* (Blyth, 1860)**

**Morphological material:** IAPG A8312 – 8313, 27.2–29.6 mm SL; Thailand: Tak Province, Salween, Moei. — CMK 5677, 6, 17.3–32.3 mm SL; Thailand: Tak Province: Amphoe Tha Song Yang, Huei Jawang, Salween drainage. Additional data from Kottelat (1990).

**Genetic material (Voucher code plus GenBank accession numbers):**

IAPG A8312: cyt b MK887016, IRBP MK886924; IAPG A8313: cyt b MK887017, IRBP MK886925.

***Schistura crocotula* Plongsesthee, Kottelat & Beamish, 2013**

**Morphological material:** IAPG A9589 – 9590, 33.8–43.2 mm SL; Thailand: Prachuap Khiri Khan Province: Krut, Nong Yaplong. — IAPG A9591, A10513 – A10518, A11507 – A11511, 32.2–44.5 mm SL; Thailand: Prachuap Khiri Khan Province: Bang Saphan, Khanan. — IAPG A10905, 17.9 mm SL; Thailand: Prachuap Khiri Khan Province: Pranburi, Pa Lao. — CMK 21984, 6, 25.0–42.6 mm SL; CMK 21985, 7, 25.8–42.2 mm SL; Thailand: Prachuap Khiri Khan Prov.: Bangsapan, Khanan River. — CMK 16458, 2, 24.9–30.4 mm SL; Thailand: Prachuap Khiri Kan Prov.: Mae Nam Pranburi, about 5 km north of Ban Pa Lao U.— CMK 26095 8 ind., 18.3–30.6 mm SL; Myanmar, Tanintharyi Region [Tenasserim]: Lenya National Park, Yai Nauk Chaung [stream]. — CMK 26192, 5, 23.4–30.5 mm S; Myanmar, Tanintharyi Region [Tenasserim]: Lenya National Park, 1900-Chaung, Yai Nauk Chaung [stream].

**Genetic material (Voucher code plus GenBank accession numbers):** IAPG A9590: cyt b MK887020, IRBP -; IAPG A9591: cyt b MK887021, IRBP MK886928; IAPG A10513: cyt b MK887024, IRBP MK886931; IAPG A10518: cyt b -, IRBP MK886932; IAPG A10905: cyt b MK887026, IRBP MK886934.

***Schistura paucifasciata* (Hora, 1929)**

**Morphological material:** BMNH 1930.3.3.4, 1, 46.6 mm SL, paratype; (photography of mouth provided by James Maclaine); aditional data from [S1], [S2], [S3].

**Genetic material**

None.

***Schistura robertsi* Kottelat, 1990**

**Morphological material:** Thailand: Ranong province: IAPG A2351-2354, 4, 30.2-34.5 mm SL; Suwan Siri waterfall (Kra Buri drainage), 10°13'N 98°45'E. — IAPG A2553, 1, 26.3 mm SL; stream Thonglang (tributary of Khlong Kapoe), 9°34'N 98°41'E. — IAPG 5247, 1, 32.4 mm SL; Amphoe Suk Samran, stream Khlong Nakha, 9°23'N 98°28'E. Thailand: Phang Nga province: ZRC 38472, holotype, 27.0 mm SL; tributary of Khlong Khao Thalu at Ban Bang Kan, 8°33'N 98°28'E, road from Phangnga to Kapong, km 22.— IAPG A2448, 1, 26.4 mm SL; small forest creek crossing road 4 about 10 km NE of city Phang Nga (Bo Saen drainage), 8°32’N 98°34‘E. — IAPG A7049 – 7051, A11002, 4, 23.1 – 30.5 mm SL; stream Kapong, river Takua Pa. — IAPG A10966 – 10968, CMK 28808, 5, (formerly IAPG A10969-10970), 28.5 – 31.4 mm SL; Amphoe Takua Pa, Khao Lak region; waterfall Sai Rung, 8°44'26"N 98°16'45"E. — CMK 22081, 2, 20.1-21.3 mm SL; small stream at Ban Kuck Nai, about 5 km east of Takua Pa, 8°53'31"N 98°24'05"E. — IAPG A2424 – A2427, 4, 19.5 – 22.7 mm SL; Sri Phang Nga NP, no details. Thailand: Phuket province: MCZ 49164, paratypes, 23, 25.3-32.3 mm SL; waterfall Ton Sai. — ZRC 49182, 1, 36.2 mm SL; waterfall Bang Poe. — ZRC 43693, 45718, 16, 12.1-32.7 mm SL; waterfall Ton Sai. — ZRC uncatalogued, 6, 25.1-41.2 mm SL; waterfall Manik. — IAPG A1667, 1, 32.0 mm SL; Phuket Island, no details. Thailand: Surat Thani province: CMK 5179, 1, 22.5 mm SL; Khlong Sok at Ban Khlong Sok. — IAPG A 4673, A4680 – A4681, A11262, A11253 – 11260, 12, 22.8 – 31.3 mm SL; stream flowing out of Khao Sok NP, 8°55'N 98°31'E. Thailand: Krabi province: IAPG A11263, 1, 32.5 mm SL; stream flowing out of Khao Phanom Bencha NP (Krabi Noi drainage), 8°12'N 98°56'E. Thailand: Nakhon Si Thammarat province: IAPG A6995, 1, 29.8 mm SL; waterfall Khao Men (Tapi drainage).

**Genetic material (Voucher code plus GenBank accession numbers):** IAPG A1667: cyt b MK886955; IRBP MK886865; IAPG A2351: cyt b MK886956; IRBP MK886866; IAPG A2352: cyt b MK886957; IRBP MK886867; IAPG A2353: cyt b MK886958; IRBP MK886868; IAPG A2424: cyt b MK886959; IRBP MK886869; IAPG A2425: cyt b MK886960; IRBP MK886870; IAPG A2426: cyt b MK886961; IRBP MK886871; IAPG A2448: cyt b MK886965; IRBP MK886875; IAPG A2553: cyt b MK886970; IRBP MK886879; IAPG A4673: cyt b MK886992; IRBP MK886901; IAPG A4680: cyt b MK886996; IRBP MK886905; IAPG A4681: cyt b MK886997; IRBP MK886906; IAPG A5247: cyt b MK887005; IRBP MK886913; IAPG A7049: cyt b MK887010; IRBP MK886918; IAPG A7050: cyt b MK887011; IRBP MK886919; IAPG A7051: cyt b MK887012; IRBP MK886920; IAPG A10966: cyt b MK887027; IRBP MK886935; IAPG A11002: cyt b MK887030; IRBP MK886938; IAPG A11262: cyt b MK887041; IAPG A11263: cyt b MK887042.

***Schistura tenebrosa* Kangrang, Page, Beamish, 2012**

**Morphological material:** High resolution photographs of holotype and details of the mouth (UF webpage); aditional data from [S4].

**Genetic material (Voucher code plus GenBank accession numbers):** UF 181418: cyt b JQ659026, IRBP -.

# *Schistura hartli*, new species

**Morphological material (non-types):** Photograph of live specimen collected with holotype.

**Genetic data(Voucher code plus GenBank accession numbers):** ZRC 61581 holotype: cyt b MK886977, IRBP MK886886; CMK 28810 paratype: cyt b MK886978, IRBP MK886887

***Schistura ataranensis*, new species**

**Morphological material (non-types):** IAPG A2560-2561, 2, 38.1-42.6 mm SL; IAPG A5062-5064, 3, 35.4-37.2 mm SL; ornamental fish trade.

**Genetic data (Voucher code plus GenBank accession numbers):** ZRC 61580 paratype: cyt b MK887043; IAPG A11005 paratype: cyt b MK887031, IRBP MK886939; IAPG 2560: cyt b MK886975, IRBP MK886884; IAPG 2561: cyt b MK886976, IRBP MK886885; IAPG 5062: cyt b MK886998, IRBP MK886907; IAPG 5063: cyt b MK886999, IRBP MK886908; IAPG 5064: cyt b MK887000, IRBP MK886909.

#### Schistura myaekanbawensis, new species

**Genetic data (Voucher code plus GenBank accession numbers):** CMK 24993 paratype: cyt b MK887022; IRBP MK886929

#### Schistura kuehnei, new species

#### Morphological material (non-types): IAPG A11261, IAPG A4672, A4674 – A4679, 8, 20.3 – 33.9 mm SL; collected with holotype.

**Genetic data (Voucher code plus GenBank accession numbers):** IAPG A4672: cyt b MK886991; IRBP MK886900; IAPG A4674: cyt b MK886993; IRBP MK886902; IAPG A4675: cyt b MK886994; IRBP MK886903; IAPG A4676: cyt b MK886995; IRBP MK886904; IAPG A11261: cyt b MK887040.

***Schistura peninsulae*, new species**

**Morphological material (non types):** Thailand: Phang Nga province: IAPG A2445-A2447, A2449-2453, A7452-A7454, 11, 17.3–34.2 mm SL; small forest creek crossing road 4 about 10 km NE of city Phang Nga (Bo Saen drainage), 8°32’N 98°34‘E. Thailand: Krabi province: IAPG A6974-A6975, 2, 29.5–30.8 mm SL; stream flowing out of Khao Phanom Bencha NP (Krabi Yai drainage), 8°14'N 98°54'E. Thailand: Surat Thani province: IAPG A4396-A4412, A9533, 18, 28.9–48.1 mm SL; Amphoe Vibhavadi, river Yan (Tapi drainage). — IAPG A11018-A11023, IAPG A11248, IAPG A11504-A11505, IAPG A11548-A11549, 11, 25.9–49.3 mm SL; stream in Amphoe Khiri Rat Nikhom (Tapi drainage). — IAPG A12010-12014, 5, 37.7–39.7 mm SL; small stream draining into Khlong Sok (Tapi drainage), 8°55`N 98°36'E. Thailand: Nakhon Si Thammarat province: IAPG A6988-6994, A7146-A7148, 10, 28.7–33.9 mm SL; waterfall Khao Men, (Tapi drainage); — IAPG A10504-10512, A11030-A11032, 11, 28.9–42.2 mm SL; waterfall Karom (Sao Thong drainage). — IAPG A11309, 1, 31.7 mm SL; Amphoe Nopphitam, waterfall Krung Ching (Klai drainage). Thailand: Trang province: IAPG A5179, 1, 36.2 mm SL; stream Khui at road 4123 (Trang drainage), 7°43’N 99°40’E. Thailand: Satung province: IAPG A6892-A6893, 2, 24.5–26.7 mm SL; Amphoe Manang, Waterfall Dao Krachan. Rangu. Thailand: Phatthalung province: IAPG A11030, 1, 35.8 mm SL; waterfall in National Park Khao Pu - Khao Ya. Malaysia: Kedah province: IAPG A4210a-A4210b, IAPG A5084-A5093, 12, 20.1–36.2 mm SL, Langkawi Island, waterfall below seven wells (Sungai Borau drainage), 6°23'N 99°40'E.

**Genetic data (Voucher code plus GenBank accession numbers):** IAPG A2445: cyt b MK886962, IRBP MK886872; IAPG A2446: cyt b MK886963, IRBP MK886873; IAPG A2447: cyt b MK886964, IRBP MK886874; IAPG A2449: cyt b MK886966, IRBP MK886876; IAPG A2450: cyt b MK886967, IRBP MK886877; IAPG A2451: cyt b MK886968, IRBP MK886878; IAPG A4210: cyt b MK886979, IRBP MK886888; IAPG A4396: cyt b MK886980, IRBP MK886889; IAPG A4397: cyt b MK886981, IRBP MK886890; IAPG A4398: cyt b MK886982, IRBP MK886891; IAPG A5084: cyt b MK887001, IRBP MK886910; IAPG A5085: cyt b MK887002, IRBP -; IAPG A5086: cyt b MK887003, IRBP MK886911; IAPG A5179: cyt b MK887004, IRBP MK886912; IAPG A6892: cyt b MK887006 IRBP MK886914; IAPG A6893: cyt b MK887007, IRBP MK886915; IAPG A6988: cyt b MK887008, IRBP MK886916; IAPG A6989: cyt b MK887009, IRBP MK886917; IAPG A7146: cyt b MK887013, IRBP MK886921; IAPG A7147: cyt b MK887014, IRBP MK886922; IAPG A7148: cyt b MK887015, IRBP MK886873; IAPG A10512: cyt b MK887023, IRBP MK886930; IAPG A11018: cyt b MK887033, IRBP MK886941; IAPG A11019: cyt b MK887034, IRBP MK886942; IAPG A11030: cyt b MK887035, IRBP MK886943; IAPG A11031: cyt b MK887036, IRBP MK886944; IAPG A11032: cyt b MK887037, IRBP MK886945; IAPG A11248: cyt b MK887039, IRBP -; IAPG A11309: cyt b MK887044, IRBP -.

**Part B) Detailed description of new species**

***Schistura ataranensis*, new species (FIGS. S1, S2)**

**Description:** A moderately elongated nemacheilid species. Predorsal body squarish in cross section or slightly depressed, caudal peduncle compressed. Body width uniform between head and pelvic-fin base, from there gradually narrowing until caudal-fin base. Caudal peduncle with pronounced dorsal and small ventral crests; depth of caudal peduncle nearly same as body depth. Depth of caudal peduncle 0.9-1.2 times in its length. Head slightly depressed, about 1.3 times broader than high. Maximum size of available material 42.6 mm SL.

Dorsal fin with 4 unbranched and 7½ branched rays; distal margin slightly convex; caudal fin slightly emarginate. Caudal fin with 8+8 branched rays, slightly emarginate. Pectoral fin with 9 rays; adpressed fin reaching midway to pelvic-fin origin. Pelvic fin with 7 rays; origin below dorsal-fin origin or slightly posterior; not reaching anal fin when adpressed; axillary lobe present. Anal fin with 3 unbranched and 5½ branched rays.

Lateral line incomplete, ending under anterior half of dorsal-fin base, with 18-31 pores. Cephalic lateral line system with 6 supraorbital pores, 3 supratemporal pores, 4+9 infraorbital pores, and 9 preoperculo-mandibular pores.

Anterior nostril pierced in front side of a tube ending in a filament reaching to anterior margin of eye. Mouth arched, 2–3 times wider than long. Lower lip with deep median interruption. Processus dentiformis present. Maxillary barbel reaching midway to base of pectoral fin. Inner rostral barbel not reaching nostril, outer rostral barbel reaching to 1/3 of length of maxillary barbel. No sexual dimorphism observed.

1.
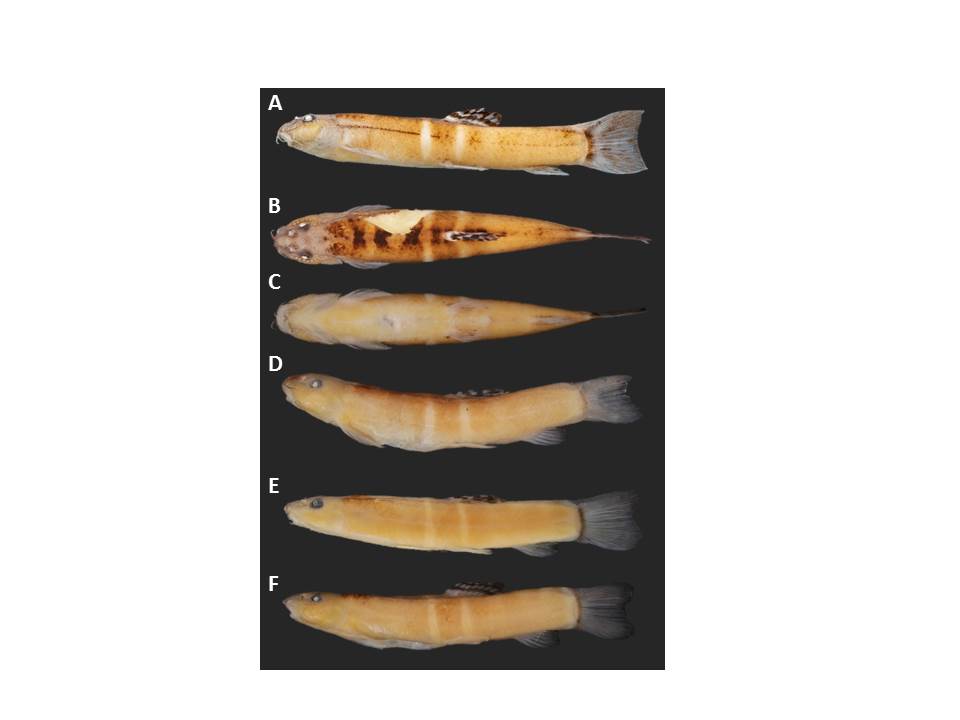


**FIG. S1.** *Schistura ataranensis*; Myanmar: Kayin province: Ataran drainage; **A-C** - ZRC 61579, holotype, 43.5 mm SL; D - ZRC 61580, paratype, 36.4 mm SL; E-F - CMK 28809, paratypes, 37.0-41.5 mm SL.


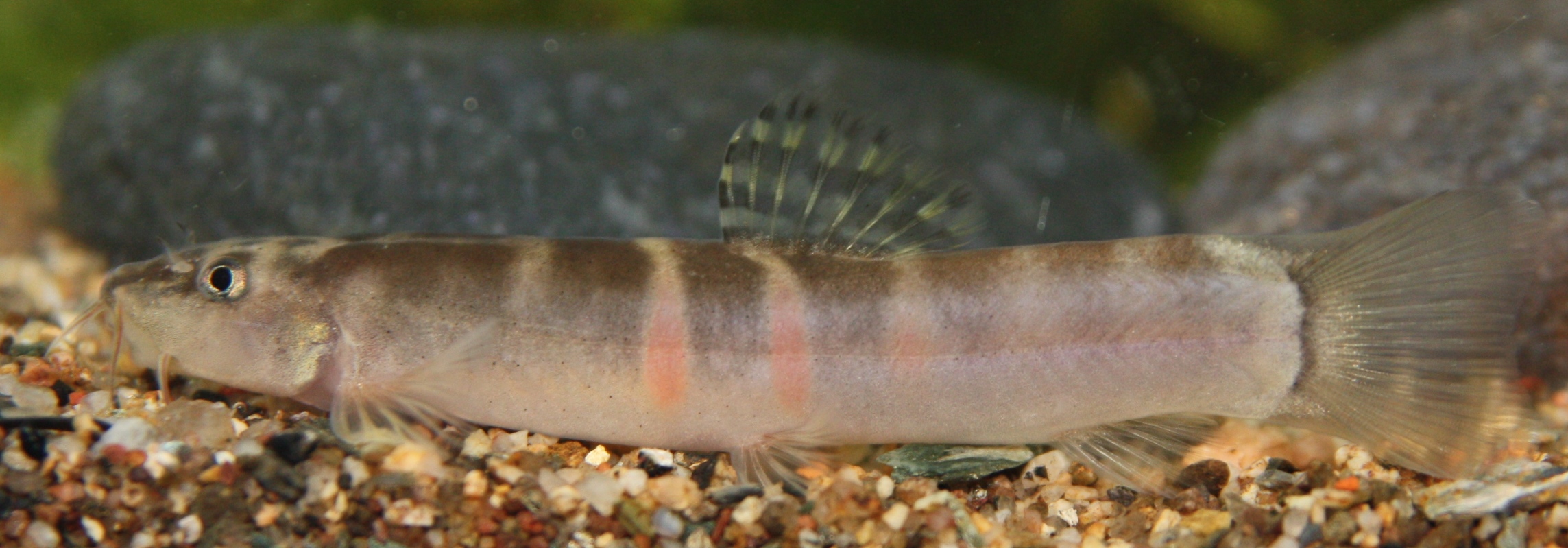


**FIG. S2.** *Schistura ataranensis*, about 39 mm SL; Myanmar: Kayin province: Ataran drainage; not preserved.

# *Schistura hartli*, new species (FIGS. S3, S4)

**Description:** A small nemacheilid species. Body elongated, slightly compressed. Largest body width behind head, from there gradually narrowing until caudal-fin base. Caudal peduncle compressed, depth only slightly less than maximum body depth. Dorsal and ventral adipose crests on caudal peduncle. Depth of caudal peduncle 0.95-1.1 times in its length. Head depressed, twice wider than deep. Snout triangular in lateral view, trapezoidal in dorsal view. Maximum known size 41.0 mm SL (but only three specimens known).

Dorsal fin with 3–4 unbranched and 7½ branched rays; distal margin straight or slightly convex. Caudal fin with 8+8 branched rays, slightly emarginate. Pectoral fin with 9 rays; adpressed fin reaching middle of distance to pelvic-fin origin. Pelvic fin with 7 rays; origin slightly in front of dorsal-fin origin, reaching behind anus; no axillary lobe. Anal fin with 3 unbranched and 5½ branched rays. Anus more or less midway between posterior extremity of pelvic-fin base and anal-fin origin.

Lateral line incomplete, ending in front of vertical through dorsal-fin origin, with 23-24 pores. Cephalic lateral line system with 6 supraorbital pores, 3 supratemporal pores, 4+9 infraorbital pores and 8 preoperculo-mandibular pores.

Anterior nostril pierced in front side of a tube ending in a filament reaching anterior margin of eye. Mouth arched. Lower lip with deep median interruption. Processus dentiformis present. Maxillary barbel reaching halfway to base of pectoral fin. Inner rostral barbel reaching at most to base of maxillary barbel. Outer rostral barbel reaching to first quarter of maxillary barbel. No sexual dimorphism observed.


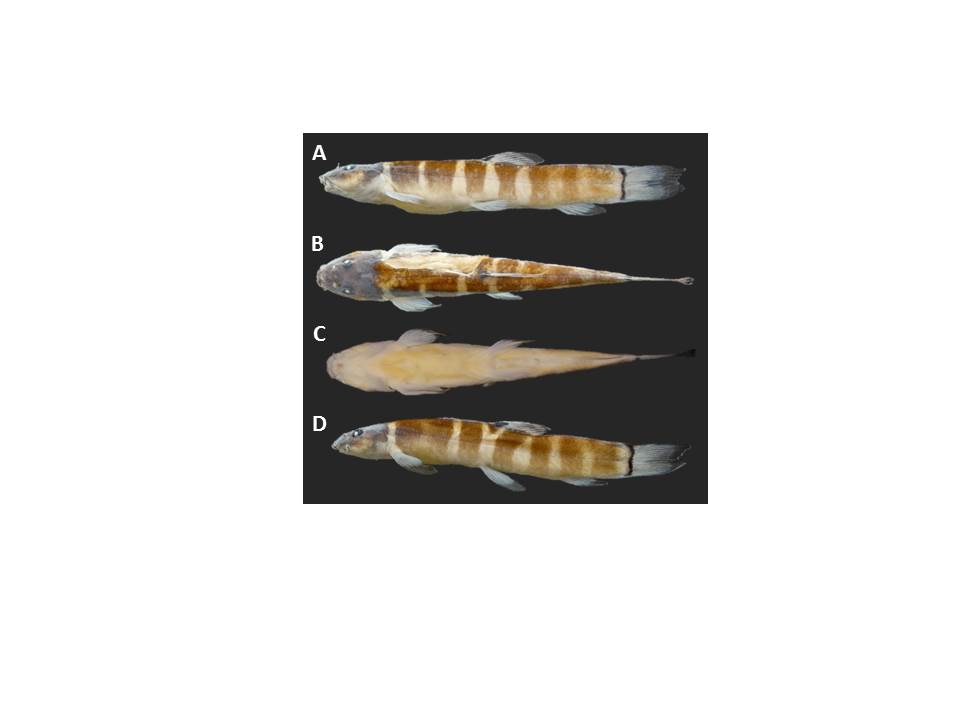


**FIG. S3.** *Schistura hartli*; Thailand: Surat Thani Province: Khao Sok NP. A-C - ZRC 61581, holotype, 41.0 mm SL; D - CMK 28810, paratype, 36.6 mm SL.


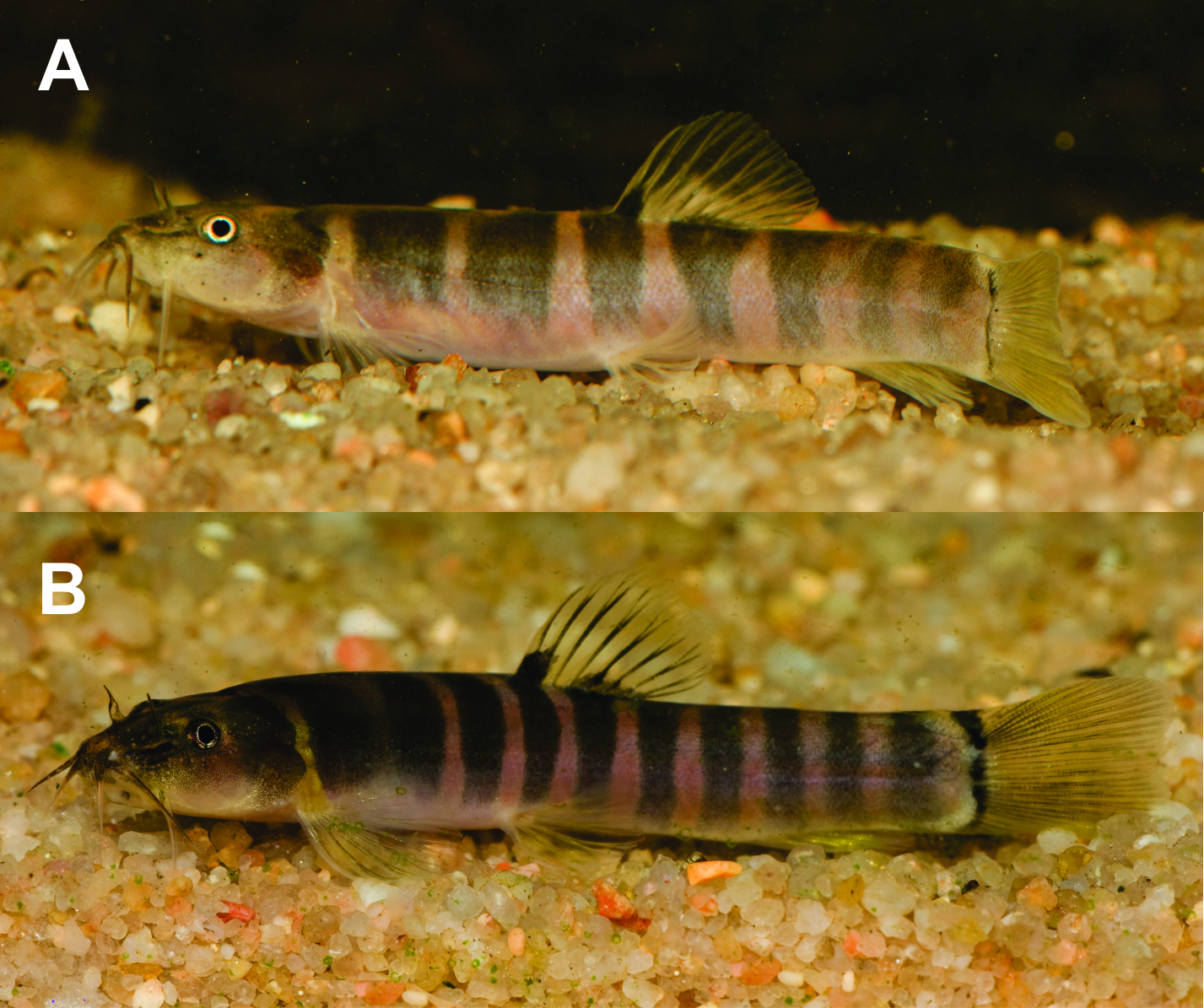


**FIG. S4.** *Schistura hartli*; Thailand: Surat Thani Province: Khao Sok NP. A – ZRC 61581, holotype, 41.0 mm SL; B – about 40 mm SL, not preserved. Photographs: A. Hartl.

#### Schistura myaekanbawensis, new species (FIG. S5)

**Description:** A small nemacheilid species. Body moderately elongated, moderately depressed in predorsal part, caudal peduncle compressed. Body widest immediately behind head, only slightly decreasing between head and posterior end of dorsal-fin base, from there gradually decreasing to caudal-fin base. Dorsal and ventral adipose crests on caudal peduncle, depth (including crests) similar to maximum body depth. Depth of caudal peduncle 0.8-0.9 times in its length. Head depressed, usually 1.5 times wider than deep. Maximum known size 29.8 mm SL.

Dorsal fin with 3-4 unbranched and 7½ branched rays (in specimens with 3 visible unbranched rays, a 4th one apparently hidden in skin anteriorly); distal margin convex. Caudal fin with 8+7 branched rays, slightly emarginate. Pectoral fin with 8 rays; adpressed fin not reaching middle of distance to pelvic fin origin. Pelvic fin with 7 rays; origin slightly in front of dorsal-fin origin, reaching behind anus; no axillary pelvic lobe. Anal fin with 3 unbranched and 5½ branched rays. Anus closer to posterior extremity of pelvic fin base than to anal-fin origin. An elongate swelling along ventral midline between anus and anal-fin origin.

Lateral line incomplete, reaching at most to 2/3 of distance between pectoral-fin base and dorsal-fin origin, with 12-18 pores. Cephalic lateral line system with 6 supraorbital pores, 3 supratemporal pores, 4 + 8-9 infraorbital and 9 preoperculo-mandibular pores.

Anterior nostril pierced in front side of a tube ending in a filament reaching anterior margin of eye. Mouth arched, 1.5-2.0 times wider than long. Lower lip with deep median interruption. Lips very thick with several folds on sides of median interruption and on each corner. Processus dentiformis present. Maxillary barbel reaching at most halfway to base of pectoral fin. Inner rostral barbel reaching to base of maxillary barbel. Outer rostral barbel reaching to midlength of maxillary barbel. Lips, barbels and tip of snout covered by unculi. No sexual dimorphism observed.

**
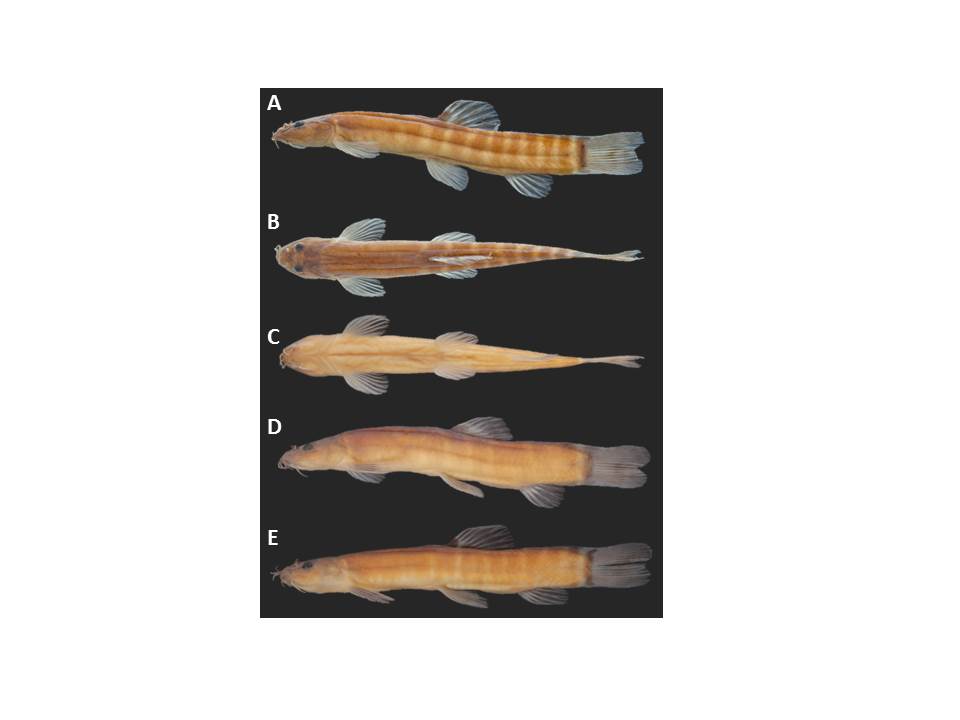
**

**FIG. S5.** *Schistura myaekanbawensis*; Myanmar: Tanintharyi Region: Tanintharyi drainage. A-C MHNG 2790.081, holotype, 29.8 mm SL; D - F CMK 24993, paratypes, 22.8 – 29.4 mm SL.

#### Schistura kuehnei, new species (FIGS. S6, S7)

**Description:** A small nemacheilid species. Body nearly round in cross section anteriorly, only slightly depressed. Body narrowing slowly from head to level of anal-fin origin, then strongly narrowing until caudal-fin base. Caudal peduncle compressed with dorsal and ventral adipose crests, therefore depth of caudal peduncle nearly same as maximum body depth. Depth of caudal peduncle 0.9-1.0 times in its length. Head depressed, usually 1.5 times wider than deep. Maximum known size 36.3 mm SL.

Dorsal fin with 4 unbranched and 7½ branched rays; distal margin slightly convex. Caudal fin with 9+8 branched rays; truncate to slightly emarginate. Pectoral fin with 9 rays; adpressed fin reaching middle of distance to pelvic-fin origin. Pelvic fin with 7 rays; origin slightly in front of dorsal-fin origin, reaching behind anus; no axillary pelvic lobe. Anus roughly midway between posterior extremity of pelvic-fin base and anal-fin origin. Lateral line incomplete, reaching at most to vertical through dorsal-fin origin, with 14-22 pores, in most specimens continuing as series of groups of three pores until caudal peduncle. Cephalic lateral line system with 6 supraorbital pores, 3 supratemporal pores, 4+9 infraorbital pores and 9 preoperculo-mandibular pores.

Anterior nostril pierced in front side of a tube ending in a filament reaching rim of eye. Mouth arched, 3-4 times wider than long. Lower lip with deep median interruption. Processus dentiformis present. Maxillary barbel reaching halfway to base of pectoral fin. Inner rostral barbel much shorter than outer one, reaching at most to base of maxillary barbel. Outer rostral barbel very long, reaching half of maxillary barbel. No sexual dimorphism observed.


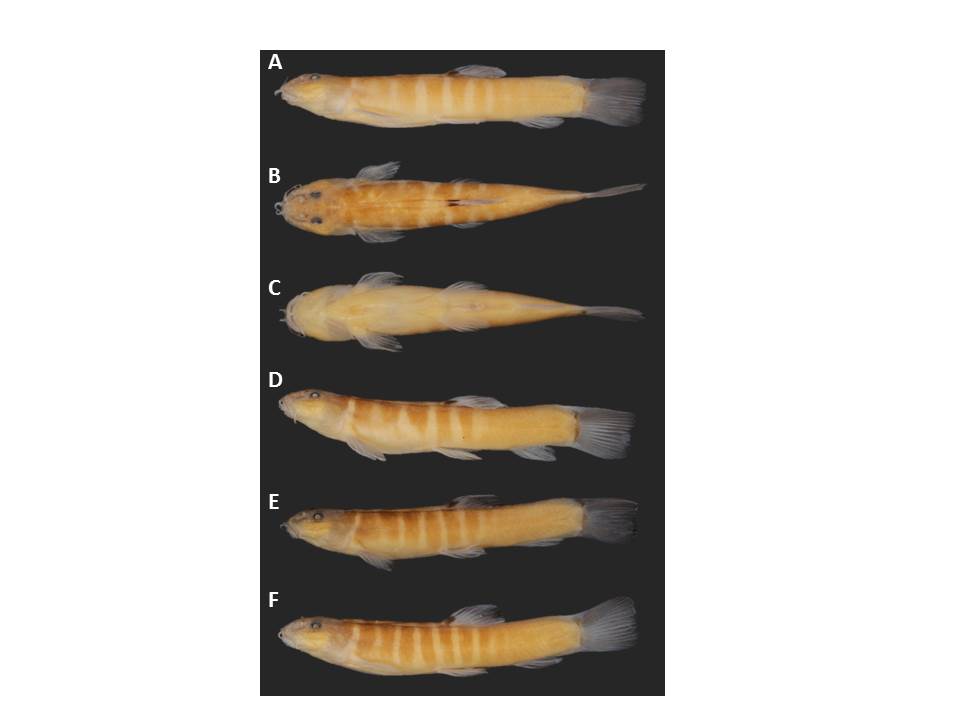


**FIG. S6.** *Schistura kuehnei*; Thailand: Surat Thani Province: Tapi drainage, Khlong Sok watershed; A-C - ZRC 61582, holotype, 37.1 mm SL; D - ZRC 61583, paratype, 31.6 mm SL; E – F – CMK 28811, paratypes, 33.9 – 35.6 mm SL.


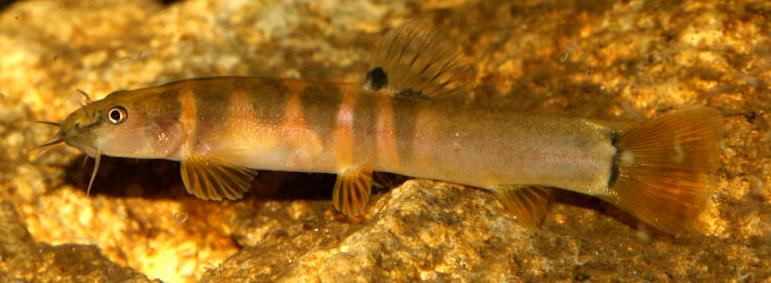


**FIG. S7.** *Schistura kuehnei*, IAPG A4671, 31.4 mm SL; Thailand: Surat Thani Province: Tapi drainage, Khlong Sok watershed; shortly after capture, right side, reversed. Photograph J. Kühne.

***Schistura peninsulae* new species (FIG. S8)**

**Description:** A small nemacheilid species. Body slightly depressed anteriorly, nearly round in cross section, width constant to pelvic-fin base, then gradually narrowing towards caudal-fin base. Caudal peduncle compressed, depth around 2/3 of body depth. Dorsal and ventral adipose crests on caudal peduncle. Depth of caudal peduncle 0.75-1 times in its length. Head depressed, usually 1.5 times wider than deep. Maximum known size 32.5 mm SL.

Dorsal fin with 3-4 unbranched and 7½ branched rays; distal margin convex. Caudal fin with 8-9 branched rays in upper lobe and 7-8 in lower lobe; truncate to emarginate. Pectoral fin with 9-10 rays; adpressed fin not reaching midway to pelvic-fin origin. Pelvic fin with 7 rays; origin in front of dorsal-fin origin, not reaching anus. No axillary pelvic lobe. Anal fin with 3 unbranched and 5½ branched rays. Anus closer to anal-fin origin than to posterior extremity of pelvic-fin base.

Lateral line incomplete, not reaching beyond tip of longest ray of adpressed pectoral fin, with 7-12 pores; in most specimens, lateral line continued by pairs of small pores every 2-5 scales; smaller than cephalic sensory pores; one pore in dorsal region of scale, one in ventral region. Cephalic lateral line system with 6 supraorbital pores, 4 supratemporal pores, 4+8 infraorbital pores and 9 preoperculo-mandibular pores.

Anterior nostril pierced in front side of a tube ending in a filament reaching anterior margin of eye. Mouth arched, 1.5-2.0 times wider than long. Lower lip with deep median interruption. Processus dentiformis present. Maxillary barbel reaching halfway to base of pectoral fin. Inner rostral barbel reaching at most to base of maxillary barbel; outer rostral barbel reaching half of maxillary barbel. No sexual dimorphism observed.


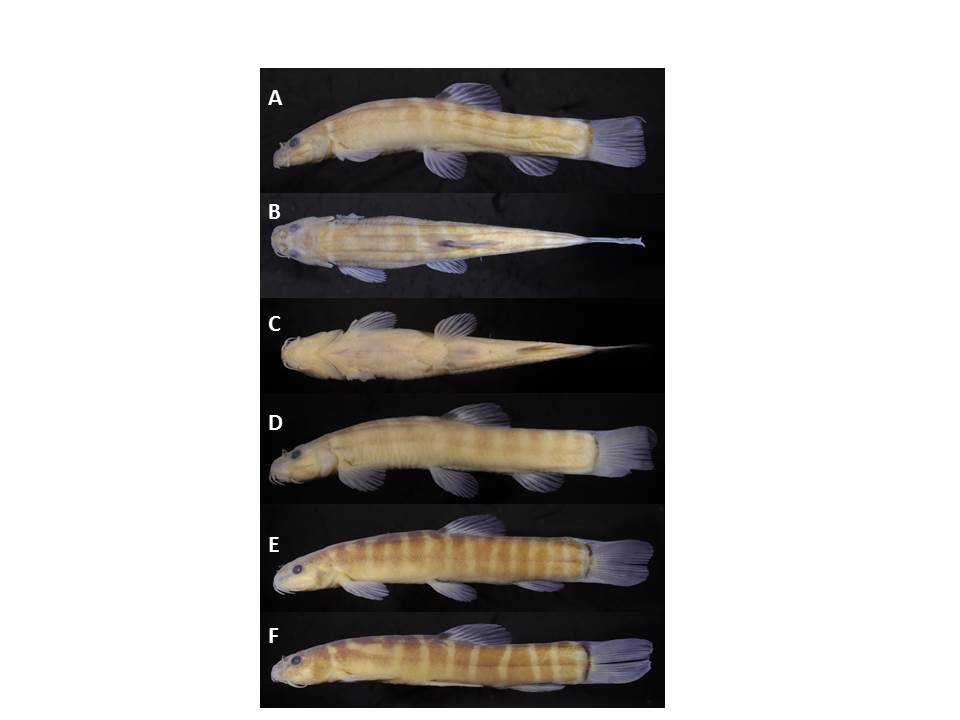


**FIG. S8.** *Schistura peninsulae*; **A-C** ZRC 61584, holotype, 35.6 mm SL; Malaysia: Kedah province: Langkawi Island: waterfall below seven wells (Sungai Borau drainage); **D** CMK 29091, 1, 38.2 mm SL; collected with holotype; **E-F** ZRC 61585, paratypes, 38.2 and 38.4 mm SL; Thailand: Nakhon Si Thammarat Province: Amphoe Chang Klang: Tapi drainage: waterfall 'Khao Mhen'.

**References**

[S1] Hora, S. L. Notes on fishes in the Indian Museum. Loaches of the Genus *Nemachilus* from Burma. *Rec. Indian Mus*. **31**:311-334 (1929).

[S2] Menon, A. G. K. The fauna of India and the adjacent countries. Pisces IV Teleostei – Cobitidae. Part 1. Homalopteridae. Zoological Survey of India, Calcutta (1987).

[S3] Kottelat, M. Indochinese nemacheilines. A revision of nemacheiline loaches (Pisces: Cypriniformes) of Thailand, Burma, Laos, Cambodia and southern Viet Nam. Pfeil, München (1990).

[S4] Kangrang, P., Page, L.M. & Beamish, W.H. *Schistura tenebrosa*, a new species of loach from the Kwai Noi River system, Mae Khlong basin, Thailand (Teleostei: Nemacheilidae). *Zootaxa* **3586**: 69-77 (2012).

**Table S1:** Additional material used for the current study; including individual code and GenBank accession numbers.

| Species | ID | Cytochrome b | IRBP 2 |
| --- | --- | --- | --- |
| Additional species of *Schistura*: |  |  |  |
| *Schistura albirostris* | KIZ 2014001499 | MG238242 | MG238356 |
| *Schistura amplizona* | KIZ2015002065 | MG238243 | MG238357 |
| *Schistura beavani* | NBFGR NV32 | GQ478448 |  |
| *Schistura bolavenensis* | A4618 | KP738575 | KP738495 |
| *Schistura bucculenta* | KIZ 20080614 | JN837654 |  |
| *Schistura callidora* | A3909 | OL191189 | OL345507 |
| *Schistura callichromus* | KIZ 2013004935 | MG238244 | MG238359 |
| *Schistura caudofurca* | KIZ 2007002414 | MG238245 | MG238360 |
| *Schistura conirostris* | KIZ 2010001442 | MG238247 | MG238362 |
| *Schistura corica* | A6945 | KP738592 | KP738512 |
| *Schistura crabro* | CMK24559 | OL191231 | OL345547 |
| *Schistura cryptofasciata* | KIZ 2004012536 | MG238250 | MG238366 |
| *Schistura desmotes* | UF183066 | MK301363 |  |
| *Schistura devdevi* | A7541 | KP738608 | KP738528 |
| *Schistura disparizona* | KIZ 2005005189 | MG238252 | MG238368 |
| *Schistura dubia* | UF181122 | MK301364 |  |
| *Schistura fasciolata* | A5301 | KP738580 | KP738500 |
| *Schistura hypsiura* | A6922 | KP738584 | KP738504 |
| *Schistura implicata* | KIZ 20130294 | MG238289 | MG238406 |
| *Schistura incerta* |  | MK361215 | KP695078 |
| *Schistura jarutanini* |  | NC_031584 |  |
| *Schistura kaysonei* |  | NC_031580 |  |
| *Schistura kloetzliae* | CMK25994_1 | OL191240 | OL345556 |
| *Schistura kongphengi* | KIZ 20150129 | MG238264 | MG238381 |
| *Schistura latidens* | KIZ 20150133 | MG238266 | MG238383 |
| *Schistura latifasciata* | KIZ 2008000127 | MG238268 | MG238385 |
| *Schistura longa* | KIZ 2010002364 | MG238272 | MG238389 |
| *Schistura macrocephalus* | KIZ 2010001595 | MG238274 | MG238391 |
| *Schistura macrotaenia* | KIZ 20100035 | JN837655 |  |
| *Schistura magnifluvis* | KIZ 2008008301 | JN837654 | MG238355 |
| *Schistura mahnerti* | A777 | OL191152 | OL345471 |
| *Schistura malaisei* | KIZ 2014005905 | MG238279 | MG238396 |
| *Schistura nicholsi* |  | DQ105202 |  |
| *Schistura notostigma* | A7519 | KP738595 | KP738515 |
| *Schistura obliquofascia* | SR3 | HM636831 |  |
| *Schistura paucicincta* | A4856 | OL191199 | OL345517 |
| *Schistura poculi* | A744 | OL191147 | OL345468 |
| *Schistura polytaenia* | KIZ 2006010328 | MG238280 | MG238397 |
| *Schistura porthos* | KIZ 2010003121 | MG238282 | MG238399 |
| *Schistura pridii* | A7548 | KP738602 | KP738522 |
| *Schistura reticulofasciata* |  | KY379150 |  |
| *Schistura rupecula* |  | AP011306 |  |
| *Schistura savona* | A7530 | KP738598 | KP738518 |
| *Schistura scaturigina* | A3925 | OL191191 | OL345509 |
| *Schistura sijuensis* | A3698 | OL191184 | OL345503 |
| *Schistura sikmaensis* | KIZ 2006010329 | JF340405 |  |
| *Schistura* sp. 'Goat Chaung' | A3006 | OL191174 | OL345493 |
| *Schistura spilota* | A958 | EF508596 |  |
| *Schistura susannae* | KIZ 20130314 | MG238288 | MG238405 |
| *Schistura thavonei* | CMK26066 | OL191243 | OL345559 |
| *Schistura tirapensis* | A3703 | OL191187 | OL345505 |
| *Schistura udomritthiruji* | A1129 | MK886954 | MK886948 |
| *Schistura vinciguerrae* | A6564 | OL191211 | OL345527 |
| *Schistura yingjiangensis* | KIZ 2014002137 | MG238294 | MG238411 |
| Other nemacheilid genera: |  |  |  |
| *Barbatula barbatula* | A8394 | KP738605 | KP738525 |
| *Lefua costata* | A6942 | KP738591 | KP738511 |
| *Mesonoemacheilus guentheri* | A6935 | KP738588 | KP738508 |
| *Mustura bella* | CMK26052 | OL191242 | OL345558 |
| *Mustura geisleri* | A1238 | OL191158 | OL345477 |
| *Mustura shanensis* | A6773 | OL191220 | OL345536 |
| *Nemacheilus binotatus* | A6926 | KP738586 | KP738506 |
| *Nemachilichthys ruppelli* | A4341 | KP738578 | KP738493 |
| *Paracanthocobitis pictilis* | A6940 | KP738589 | KP738509 |
| *Paracanthocobitis zonalternans* | A5331 | KP738582 | KP738502 |
| *Paraschistura montana* | NBFGR NR20 | FJ711438 |  |
| *Petruichthys brevis* | A4185 | KP738572 | KP738492 |
| *Physoschistura brunneana* | A578 | OL191138 | OL345460 |
| *Physoschistura pseudobrunneana* | A1356 | OL191163 | OL345482 |
| *Physoschistura rivulicola* | A6670 | OL191214 | OL345530 |
| *Physoschistura shuangjiangensis* | A2999 | OL191173 | OL345492 |
| *Physoschistura* sp. | A7545 | KP738600 | KP738520 |
| *Pteronemacheilus luciodorsum* | A8465 | OL191215 | OL345531 |
| *Pteronemacheilus meridionalis* | A5851 | OL191208 | OL345524 |
| *Seminemacheilus lendlii* | A4834 | KP738578 | KP738498 |
| *Triplophysa grahami* | A1663 | MK608125 | MT53672 |
| *Triplophysa siluroides* | A1797 | MT536720 | MT536723 |
| *Triplophysa strauchi* | A11496 | MT536721 | MT536724 |
|  |  |  |  |
| Cobitid outgroup: |  |  |  |
| *Cobitis taenia* | A1860 | EF508508 | MK608315 |
|  |  |  |  |
